# Allosteric Activation of 15‐Lipoxygenase‐1 by Boswellic Acid Induces the Lipid Mediator Class Switch to Promote Resolution of Inflammation

**DOI:** 10.1002/advs.202205604

**Published:** 2022-12-25

**Authors:** Friedemann Börner, Simona Pace, Paul M. Jordan, Jana Gerstmeier, Mario Gomez, Antonietta Rossi, Nathaniel C. Gilbert, Marcia E. Newcomer, Oliver Werz

**Affiliations:** ^1^ Department of Pharmaceutical/Medicinal Chemistry Institute of Pharmacy Friedrich‐Schiller‐University Jena Philosophenweg 14 07743 Jena Germany; ^2^ Evonik Operations GmbH Kirschenallee 45 64293 Darmstadt Germany; ^3^ Department of Pharmacy School of Medicine and Surgery University of Naples Federico II Via D. Montesano 49 Naples I‐80131 Italy; ^4^ Department of Biological Sciences Louisiana State University 202 Life Science Building Baton Rouge LA 70803 USA

**Keywords:** boswellic acid, inflammation, lipid mediators, lipoxygenases

## Abstract

Specialized pro‐resolving mediators (SPM), primarily produced in innate immune cells, exert crucial bioactions for resolving inflammation. Among various lipoxygenases (LOX), 15‐LOX‐1 is key for SPM biosynthesis, but cellular activation principles of 15‐LOX‐1 are unexplored. It was shown that 3‐*O*‐acetyl‐11‐keto‐*β*‐boswellic acid (AKBA) shifts 5‐LOX regiospecificity from 5‐ to 12‐lipoxygenation products. Here, it is demonstrated that AKBA additionally activates cellular 15‐LOX‐1 via an allosteric site accomplishing robust SPM formation in innate immune cells, particularly in M2 macrophages. Compared to ionophore, AKBA‐induced LOX activation is Ca^2+^‐ and phosphorylation‐independent, with modest induction of 5‐LOX products. AKBA docks into a groove between the catalytic and regulatory domains of 15‐LOX‐1 interacting with R98; replacement of R98 by alanine abolishes AKBA‐induced 15‐LOX product formation in HEK293 cells. In zymosan‐induced murine peritonitis, AKBA strikingly elevates SPM levels and promotes inflammation resolution. Together, targeted allosteric modulation of LOX activities governs SPM formation and offers new concepts for inflammation resolution pharmacotherapy.

## Graphical abstract

A boswellic acid is discovered as a molecular switch enabling innate immune cells to block formation of pro‐inflammatory eicosanoids and to generate specialized pro‐resolving mediators by modulation of lipoxygenase isoforms at allosteric sites. These findings advance inflammation resolution pharmacology and may give direction for development of new therapeutics for treating diseases connected to unresolved inflammation.

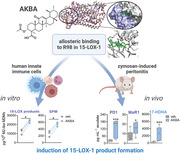

## 1 Introduction

The acute inflammatory response is crucial for host defense to protect the body from invading microbes or physical injuries and is pivotal for tissue repair and return to homeostasis.^[^ ^1^ ^]^ Natural physiological control of inflammation, especially effective resolution programs, are therefore essential to avoid the progression to excessive inflammatory disease states.^[^ ^2^ ^]^ Various environmental and lifestyle factors can promote chronic systemic inflammation, contributing to widespread diseases including obesity, atherosclerosis, type 2 diabetes, autoimmune, and neurodegenerative ailments, which are the leading causes of disability and mortality in the developed world.^[^ ^2^, ^3^ ^]^ Accordingly, therapeutically targeting inflammation by inhibition of the inflammatory mediators and concomitant alteration of signaling is of great interest. However, inflammatory mediators contribute to many biological processes, including the functioning of the nervous system, tissue homeostasis, metabolism, and thermogenesis,^[^ ^4^ ^]^ as well as resolution of inflammation and tissue restoration.^[^ ^5^ ^]^ Classic inhibitors that block the production of pro‐inflammatory mediators can simultaneously impair the resolution phase of inflammation by inhibiting the enzymes common to both biosynthetic pathways in the production of pro‐ and anti‐inflammatory lipid mediators (LMs).^[^ ^2^ ^]^

**Figure 1:**
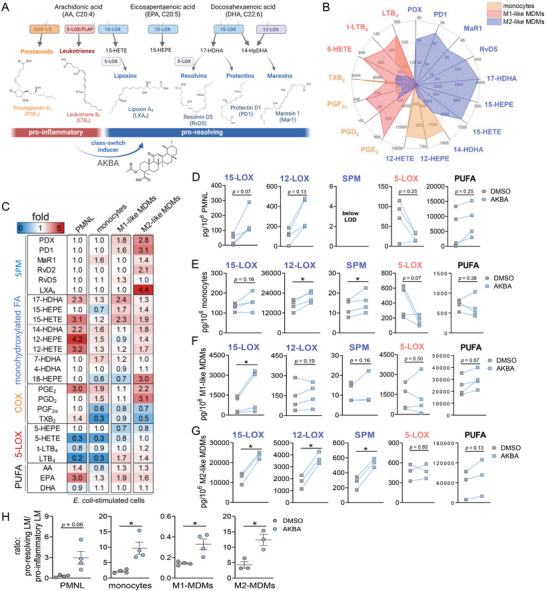
Modulation of lipid mediator profiles in activated 5‐LOX‐expressing immune cells by AKBA. A) Simplified biosynthetic scheme of lipid mediator (LM) formation from PUFAs involving COX and LOX enzymes and modulation by AKBA. B) Human monocytes, M1‐MDMs, and M2‐MDMs were stimulated with *E. coli* (O6:K2:H1; ratio 1:50) for 90 min at 37 °C. Formed LM were isolated from the supernatants by SPE and analyzed by UPLC‐MS‐MS and shown as means in a radar plot, *n* = 3–4. C–H) Human PMNL, monocytes, M1‐MDMs, and M2‐MDMs were preincubated with 10 µm AKBA or vehicle (0.1% DMSO) for 15 min before stimulation with *E. coli* (O6:K2:H1; ratio 1:50) for 90 min at 37 °C. Formed LM were isolated from the supernatants by SPE and analyzed by UPLC‐MS‐MS. C) Heatmap showing the fold changes in LM formation for AKBA‐ versus vehicle‐pretreated cells stimulated with *E. coli, n* = 3 – 4. D–G) Data are given as pg/10^6^ cells *n* = 3 – 4 separate donors, each. D) PMNL, E) monocytes, F) M1‐MDMs, and G) M2‐MDMs; bdata where values were below the detection limit (LOD, ≤3 pg/10^6^ cells) are indicated “below LOD.” Statistical analysis was done by ratio‐paired Student's *t*‐test, *p < 0.05, AKBA versus vehicle control. 15‐LOX products include 17‐HDHA, 15‐HETE, and 15‐HEPE; 12‐LOX products include 14‐HDHA, 12‐HETE, and 12‐HEPE; SPM include PD1, PDX, RvD2, RvD5, and MaR1; 5‐LOX products include t‐LTB_4_, LTB_4_, 5‐HETE, and 5‐HEPE; COX products include PGE_2_, PGD_2_, TXB_2_, and PGF_2*α*_, and PUFA include AA, EPA, and DHA. H) The ratio of pro‐resolving LM (sum of 15‐LOX products, 12‐LOX‐products, and SPMs) against pro‐inflammatory LM (sum of 5‐LOX and COX‐products) were shown in scatter dot plots for each distinct cell type. Statistical analysis was done by paired Student's *t*‐test, *p < 0.05, AKBA versus vehicle control.

LMs are produced from polyunsaturated fatty acids (PUFAs), such as arachidonic acid (AA, 20:4, *ω*‐6), eicosapentaenoic acid (EPA, 20:5, *ω*‐3), and docosahexaenoic acid (DHA, 22:6, *ω*‐3) primarily by cyclooxygenases (COX) and lipoxygenases (LOX) in complex interconnected biosynthetic networks (**Figure** **1**A), and crucially contribute to the onset and progression of inflammation and its resolution.^[^ ^5^, ^6^ ^]^ While COX‐derived prostaglandins (PGs) and 5‐LOX‐derived leukotrienes (LTs) are produced from AA and foster inflammation,^[^ ^6b^ ^]^ the specialized pro‐resolving mediators (SPMs) are generated mainly from EPA and DHA, and to a lesser extent from AA, possessing inflammation‐resolving activities^[^ ^5^, ^6^, ^7^ ^]^ (Figure 1A). SPMs encompasses lipoxins (LX), resolvins (RV), protectins (PD), and maresins (MaR) which possess a huge potential as a new direction for therapy of diseases with an unresolved inflammatory component: they promote endogenous resolution programs by counteracting excessive neutrophil infiltration and by stimulating macrophage‐mediated clearance of microbes, apoptotic polymorphonuclear leukocytes (PMNL), and cellular debris, as well as by suppressing pro‐inflammatory mediator production.^[^ ^7^ ^]^ In fact, while the pro‐inflammatory PGs are the prime targets of non‐steroidal anti‐inflammatory drugs (NSAIDs) in traditional inflammation pharmacotherapy,^[^ ^8^ ^]^ novel therapeutic strategies currently pursue the initiation of endogenous resolution programs, for example, by SPMs as immunoresolvents.^[^ ^7^, ^9^ ^]^

SPM biosynthesis is mainly driven by 12/15‐LOXs, partially in conjunction with the LT‐forming 5‐LOX.^[^ ^10^ ^]^ Favorable manipulation of these complex LOX/LM networks requires smart pharmacological concepts such as accomplishing the switch from pro‐inflammatory PG and LT toward anti‐inflammatory SPMs. In light of these considerations, we recently discovered that 3‐*O*‐acetyl‐11‐keto‐*β*‐boswellic acid (AKBA), an active principle component of the traditional anti‐inflammatory remedy frankincense, modulates 5‐LOX via an allosteric site, invoking a shift in the regiospecificity toward a 12/15‐lipoxygenating enzyme.^[^ ^11^ ^]^ SPM generation requires oxygenations of PUFAs by 12/15‐LOXs,^[^ ^12^ ^]^ and indeed, AKBA increased the levels of 12‐LOX products and SPMs in HEK293 cells (devoid of 12/15‐LOX) expressing solely 5‐LOX.^[^ ^11^ ^]^ Here, we studied the ability of AKBA to shift the formation of LTs to SPM in human innate immune cells and in zymosan‐induced murine peritonitis, a self‐limited in vivo inflammation model. Our data reveal that AKBA not only alters 5‐LOX regiospecificity but also robustly activates 15‐LOX for SPM biosynthesis by unique mechanisms, that is, allosteric modulation of cellular 15‐LOX‐1 activity. Conclusively, a new pharmacological concept for advancing the resolution phase of inflammation is the thoughtful manipulation of LOX activities by switching from the production of pro‐inflammatory LTs to anti‐inflammatory SPMs with “class‐switching” compounds.

## 2 Results

### 2.1 AKBA Modulates LM Signature Profiles in *Escherichia coli*‐Activated Human Innate Immune Cells

PMNL, monocytes, and macrophages are key innate immune cells of the inflammatory response and express various LOXs and COX enzymes to generate broad spectra of LM.^[^ ^5^, ^6^ ^]^ We studied how AKBA impacts the LM signature profiles in these innate immune cells by performing comprehensive LM metabololipidomics using UPLC‐MS‐MS. Human PMNL, monocytes, and monocyte‐derived macrophages (MDMs) of M1 or M2 phenotype were pretreated (15 min) with 10 µm AKBA before stimulation with pathogenic *Escherichia coli* (*E. coli*) (O6:K2:H1; ratio 1:50, 90 min) as a suitable stimulus.^[^ ^13^ ^]^ Figure 1B and Table S1, Supporting Information, reveal cell‐type‐specific LM profiles in these *E. coli*‐activated leukocytes, where monocytes and M1‐MDMs dominate in the formation of 5‐LOX and COX products, while M2‐MDMs generate substantial 12/15‐LOX‐derived products and SPM. Interestingly, AKBA elevated the levels of 15‐LOX and 12‐LOX products in all cell types (Figure 1C–G and Table S1, Supporting Information) and increased formation of SPMs (i.e., PDs, MaR1, RVs, and LXA_4_), especially in M2‐MDMs but with minor effects in PMNL (Figure 1C,G and Table S1, Supporting Information). In contrast, AKBA decreased 5‐LOX‐derived LTs and related products in these cells, but not so in M2‐MDMs, while liberation of AA, EPA, and DHA was not significantly (*p* < 0.05) affected (Figure 1C–G and Table S1, Supporting Information). Thus, AKBA provokes a shift in *E. coli*‐activated leukocytes from pro‐inflammatory LTs toward 12/15‐LOX products and related inflammation‐resolving SPMs (Figure 1H).

### 2.2 AKBA Evokes Robust Formation of SPM and 12/15‐LOX‐Derived LM in M2‐MDM

**Figure 2:**
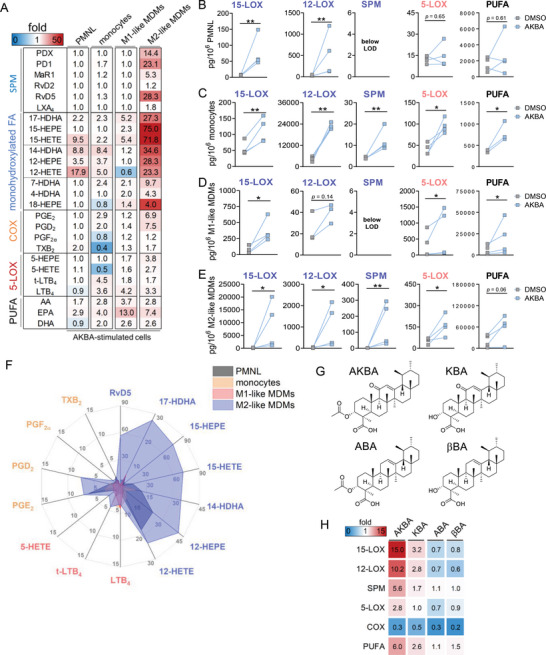
Induction of lipid mediator formation in unstimulated 5‐LOX‐rich immune cells by AKBA and related BAs. A–F) Human PMNL, monocytes, M1‐MDMs, and M2‐MDMs (10^6^ each) were incubated with 10 µm AKBA or vehicle (0.1% DMSO) for 90 min at 37 °C. Formed LM were isolated from the supernatants by SPE and analyzed by UPLC‐MS‐MS. A) Heatmap showing the fold changes in LM formation for AKBA‐ versus vehicle‐treated cells, *n* = 4. B–E) Data are means ± S.E.M. given in pg/10^6^ cells of *n* = 4 separate donors, each; data where values were below the detection limit (LOD, ≤3 pg/10^6^ cells) are indicated “below LOD.” C) PMNL, D) monocytes, E) M1‐MDMs, and F) M2‐MDMs. 15‐LOX products include 17‐HDHA, 15‐HETE, and 15‐HEPE; 12‐LOX products include 14‐HDHA, 12‐HETE, and 12‐HEPE; SPM include PD1, PDX, RvD2, RvD5, and MaR1; 5‐LOX products include t‐LTB_4_, LTB_4_, 5‐HETE, and 5‐HEPE; COX products include PGE_2_, PGD_2_, TXB_2_, and PGF_2*α*_; PUFA include AA, EPA, and DHA. F) Radar plot showing the ‐fold increase for selected LM formed by cells after AKBA treatment compared to vehicle controls. G) Chemical structures of natural *β*‐BAs. H) Structure–activity relationship of four BAs in M2‐MDMs. 2 × 10^6^ cells were treated with BAs (10 µm each) for 180 min at 37 °C. Formed LM were isolated from the supernatants by SPE and analyzed by UPLC‐MS/MS. Data are shown as a heatmap of different LM groups representing the ‐fold change of BA‐treated versus vehicle‐treated cells, *n* = 3 separate donors.

AKBA may not only modulate agonist‐induced LOX product formation but could also act as elicitor of LM biosynthesis. In fact, when cells were simply exposed to AKBA (10 µm) for 90 min, we found substantial formation of 12/15‐LOX products, including SPMs (**Figure** **2**A–F and Table S2, Supporting Information), especially in M2‐MDMs (Figure 2E) that express abundant 15‐LOX‐1.^[^ ^13b^ ^]^ Thus, AKBA elevated PD1 and RvD5 levels up to 23‐ and 28‐fold, along with 23‐ up to 75‐fold increase of the 12‐/15‐LOX‐derived 12‐HETE, 12‐HEPE, 14‐HDHA, 15‐HETE, 15‐HEPE, and 17‐HDHA in M2‐MDMs (Figure 2A,F and Table S2, Supporting Information). 3‐(4,5‐dimethylthiazol‐2‐yl)‐2,5‐diphenyltetrazolium bromide (MTT) assays or lactate dehydrogenase (LDH) release analyses revealed no detrimental effects of AKBA (10 µm) on the viability of M2‐MDMs (Figure S1, Supporting Information). COX‐ and 5‐LOX‐derived products were moderately elevated, if at all, by AKBA, regardless of the cell/phenotype and despite highly elevated SPM levels (Figure 2A and Table S2, Supporting Information). Radar plots of the LM profile of M2‐MDMs confirm potent and selective induction of 12/15‐LOX activity by AKBA versus resting cells (Figure 2F). Also, AKBA induced the release of AA, EPA, and DHA in all cells investigated (Figure 2A and Table S2, Supporting Information), thus, increasing the substrate levels for LM production. We tested three other *β*‐BAs contained in frankincense^[^ ^14^ ^]^ that lack the 11‐keto moiety and/or the 3‐O‐acetyl group, that is, KBA, ABA, and *β*BA (Figure 2G). Side‐by‐side comparison for induction of 12/15‐LOX product formation in M2‐MDMs revealed AKBA as the most potent derivative, while KBA had minimal effects, and ABA and *β*BA were inactive (Figure 2H), suggesting stringent SAR in which the 11‐keto moiety is a prerequisite. None of these BAs (10 µm) caused cytotoxicity in M2‐MDMs (Figure S2A, Supporting Information).

### 2.3 AKBA‐Evoked SPM Formation in M2‐MDM Coincides with 15‐LOX‐1 Activation

**Figure 3:**
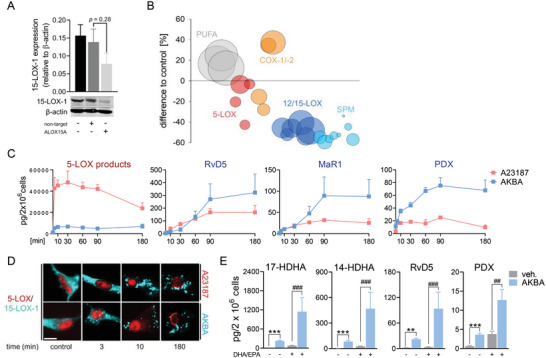
AKBA elevates 12/15‐LOX products involving 15‐LOX‐1 in M2‐MDM. A,B) MDMs (2 × 10^6^ cells mL^−1^) differentiated from human monocytes for 6 days with M‐CSF were transfected with non‐target or ALOX15A siRNA during polarization with IL‐4 (20 ng mL^−1^) for 48 h. A) Western blot analysis of 15‐LOX‐1; *β*‐actin served as reference protein for normalization after densitometric analysis, *n* = 4; Student´s unpaired *t*‐test. B) LM profile of ALOX15A siRNA‐treated M2‐MDMs after exposure to AKBA (10 µm) for 180 min, *n* = 4. Data are shown as bubble blot in % of non‐target siRNA of LM summarized in groups: 12/15‐LOX products include 17‐HDHA, 15‐HETE, 15‐HEPE, 14‐HDHA, 12‐HETE, and 12‐HEPE; SPM include PD1, PDX, RvD2, RvD5, and MaR1; 5‐LOX products include t‐LTB_4_, LTB_4_, 5‐HETE, and 5‐HEPE; COX products include PGE_2_, PGD_2_, TXB_2_, and PGF_2*α*_; PUFA include AA, EPA, and DHA. C) Kinetic of LM formation in M2‐MDMs after incubation with 10 µm AKBA or 2.5 µm A23187 in the presence of AA, EPA, and DHA (1 µm each). After the indicated time points, formed LM were isolated from the supernatants by SPE and analyzed by UPLC‐MS‐/MS. Data are shown as means ± S.E.M. given in pg/2 × 10^6^ cells for representative SPM and for the sum of 5‐LOX products, including t‐LTB_4_, LTB_4_, 5‐HETE, and 5‐HEPE; *n* = 3 separate donors. D) Subcellular redistribution of 5‐LOX and 15‐LOX‐1 in M2‐MDMs after exposure to AKBA (10 µm) or A23187 (2.5 µm) for indicated time points. Cells were fixed, permeabilized, and incubated with antibodies against 5‐LOX (red) and 15‐LOX‐1 (cyan‐blue); scale bar = 10 µm. Results shown for one single cell are representative for ≈100 individual cells analyzed in *n* = 3 independent experiments with separate donors, each. E) M2‐MDMs where co‐incubated with or without 3 µg mL^−1^ of DHA and EPA, each, and with AKBA (10 µm) or vehicle (0.1% DMSO) for 180 min at 37 °C. Formed LM were isolated from supernatants by SPE and analyzed by UPLC‐MS‐MS. Data are means ± S.E.M., given in pg/2 × 10^6^ cells, *n* = 4 independent experiments. For statistical analysis, data were log‐transformed, one‐way analysis of variance (ANOVA) with Tukey's test, **p < 0.01, ***p < 0.001 vehicle control versus AKBA; #*p* < 0.05, ##*p* < 0.01 vehicle + supplement versus AKBA + supplement.

Human M2‐MDMs contain abundant 15‐LOX‐1, a key enzyme in SPM biosynthesis, and to a much lesser degree the 15‐LOX‐2.^[^ ^15^ ^]^ To study if the 15‐LOX‐1 isoform is responsible for the enhanced SPM formation evoked by AKBA, we silenced the ALOX15A gene during M2‐MDMs polarization using small interfering RNA (siRNA) and confirmed selective depletion of the 15‐LOX‐1 protein by Western Blot (**Figure** **3**A and Figure S3, Supporting Information). 15‐LOX‐1‐depleted M2‐MDMs generated much less SPM and other 15‐LOX‐derived LM in response to AKBA versus cells that received non‐target siRNA, while COX/5‐LOX product formation did not differ between the two, as expected (Figure 3B and Table S3, Supporting Information).

We next compared the temporal LM formation evoked by AKBA with that obtained with Ca^2+^ ionophore A23187, a classical agonist of LM biosynthesis, in the presence of exogenous AA, EPA, and DHA (to ensure substrate supply‐independent LOX activities). Exposure of M2‐MDMs to 2.5 µm A23187 caused rapid and massive generation of 5‐LOX products within 3–5 min, but 12/15‐LOX products and SPMs were comparably much less generated and required longer incubation times (Figure 3C). In contrast, AKBA evoked mainly the formation of 12/15‐LOX products in M2‐MDMs peaking at 90 min, surpassing the efficiency of A23187 in this respect, but with overall moderate 5‐LOX product levels (Figure 3C). Thus, while A23187 rapidly elicits mainly 5‐LOX products in M2‐MDMs, AKBA selectively and substantially induces formation of 12/15‐LOX‐derived LMs in a rather delayed manner.

For cellular product formation, 15‐LOX‐1 requires translocation from the cytosol to a membrane to access liberated PUFA provided by phospholipases (PL)A_2_.^[^ ^13b^ ^]^ Since AKBA indeed elevated the AA, EPA, and DHA levels, we studied the role cytosolic PLA_2_ *α* as potential candidate.^[^ ^16^ ^]^ AKBA induced some minor release of ^3^H‐AA from pre‐labeled M1‐ and M2‐MDM but compared to A23187, this release was weak and only slightly suppressed by the cPLA_2_ *α* inhibitor RSC3388, excluding a major role of cPLA_2_ *α* (Figure S2B, Supporting Information). AKBA (10 µm) caused 15‐LOX‐1 redistribution within 3–10 min in M2‐MDMs where the enzyme remained up to 180 min; such temporal 15‐LOX‐1 redistribution was also induced by A23187 (Figure 3D). In agreement with induction of 15‐LOX product formation, KBA induced 15‐LOX‐1 redistribution while ABA and BA failed in this respect (Figure S2C, Supporting Information). Since AKBA induced release of AA, EPA, and DHA (see Figure 2A and Table S2, Supporting Information), elevated SPM/15‐LOX product formation may be simply due to increasing the availability of free PUFA. Supplementation of M2‐MDMs with ample amounts of exogenous EPA and DHA (3 µg mL^−1^, each) increased formation of representative SPM (PDX and RvD5) and 12/15‐LOX products, but much less efficiently than AKBA (Figure 3E and Table S4, Supporting Information). Importantly, the stimulatory effects of AKBA were not only evident in the presence of exogenous EPA/DHA but very striking with synergistic elevations (Figure 3E and Table S4, Supporting Information), implying that AKBA both promotes the release of endogenous PUFA and stimulates 15‐LOX‐1 activity.

### 2.4 AKBA Induces 15‐LOX‐Product Formation in M2‐MDM Independent of Ca^2+^ and MAPK

**Figure 4:**
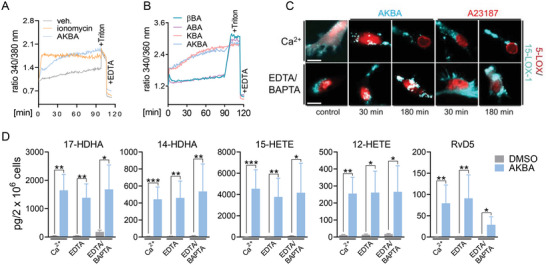
15‐LOX‐1 activation by AKBA in M2‐MDMs is insensitive to Ca^2+^. A,B) Analysis of [Ca^2+^]_i_ in Fura‐2/AM‐loaded M2‐MDMs. Cells were incubated with boswellic acids (10 µm each), 1 µm ionomycin, or vehicle (0.1% DMSO) for up to 90 min at 37 °C. Data are shown as ratio of absorbance at 340/380 nm reflecting [Ca^2+^]_i_; *n* = 4–6 independent experiments with separate donors. C,D) Human M2‐MDMs seeded in PBS containing Ca^2+^ (1 mm), EDTA (0.5 mm), or EDTA (0.5 mm) plus BAPTA/AM (20 µm) were incubated with AKBA (10 µm) or vehicle (0.1% DMSO) at 37 °C. C) After the indicated times, the subcellular redistribution of 5‐LOX and 15‐LOX‐1 in M2‐MDM after treatment with AKBA (10 µm), A23187 (2.5 µm), or vehicle (0.1% DMSO) was analyzed; cells were fixed, permeabilized, and incubated with antibodies against 5‐LOX (red) and 15‐LOX‐1 (cyan‐blue); scale bars = 10 µm. Results shown for one single cell are representative for ≈100 individual cells analyzed, *n* = 3, separate donors, each. D) After 180 min, formed LM were extracted from the supernatants by SPE and analyzed by UPLC‐MS‐MS. Data are shown as pg/2 × 10^6^ cells; *n* = 3 separate donors. For statistical analysis data were log‐transformed, *p < 0.05, **p < 0.01, ***p < 0.001 AKBA versus vehicle control; unpaired Student's *t*‐test.

In general, LOX product formation requires activation of PLA_2_ enzyme(s) for PUFA release, subcellular LOX redistribution to access the PUFA substrate, and LOX activation to oxygenate the PUFA. Historically, these processes are thought to require Ca^2+^ and/or phosphorylation by MAPK, depending on the stimulus.^[^ ^16^, ^17^ ^]^ Thus, stimuli that evoke LOX product formation like ionophore or bacterial exotoxins increase intracellular Ca^2+^ ([Ca^2+^]_i_) and/or activate MAPK.^[^ ^15^, ^16^, ^17^ ^]^ AKBA and also KBA (10 µm, each), but not ABA and *β*BA, continuously elevated [Ca^2+^]_i_ in M2‐MDMs within ≈90 min to a similar extent as ionomycin, although the latter caused a more rapid response (maximal [Ca^2+^]_i_ after ≈5 min, **Figure** **4**A,B); similar effects were evident in M1‐MDMs (Figure S4, Supporting Information). However, in contrast to ionophore or bacterial exotoxins,^[^ ^13^, ^18^ ^]^ depletion of intra‐ and extra‐cellular Ca^2+^ in M2‐MDMs by BAPTA/AM and EDTA, respectively, neither abolished AKBA‐induced 15‐LOX subcellular redistribution (Figure 4C) nor LM formation (Figure 4D and Table S5, Supporting Information), suggesting that AKBA activates 15‐LOX‐1 in a Ca^2+^‐independent manner. AKBA did not markedly affect activation (= phosphorylation) of p38 MAPK, ERK‐1/2, JNK, or Akt in M2‐MDMs (Figure S5A, Supporting Information), and pharmacological inhibition of these MAPKs (skepinone‐L for p38 MAPK, U0126 for ERK‐1/2, SP600125 for JNK), Akt (LY294002), or multiple PKs using staurosporine failed to prevent AKBA‐induced SPM and 12/15‐LOX product formation (Table S6, Supporting Information). Finally, AKBA did not change the expression of major LM‐biosynthetic enzymes and proteins in M1‐ and M2‐MDM treated with this BA (Figure S5B, Supporting Information). Taken together, AKBA appears to activate 15‐LOX‐1 in M2‐MDM through a unique mechanism that is independent of the typical Ca^2+^ and MAPK triggers.

### 2.5 AKBA Directly Activates Cellular 15‐LOX and Docks into Models of 15‐LOX‐1

**Figure 5:**
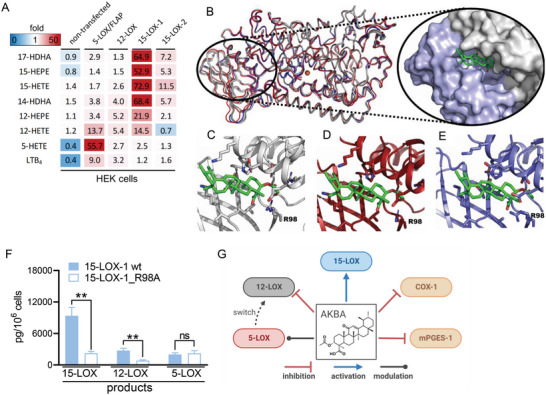
AKBA activates 15‐LOX for LM formation in transfected HEK293 cells and docks into h15‐LOX‐1. HEK293 cells (stably transfected with either 5‐LOX/FLAP, 12‐LOX, 15‐LOX‐1, or 15‐LOX‐2, as indicated) were incubated with vehicle (0.1% DMSO) or AKBA (25 µm) for 180 min in PBS plus 1 mm Ca^2+^. Supernatants were subjected to SPE and UPLC‐MS/MS analysis of formed LM, *n* = 3. A) Data are shown as means in a heatmap representing the ‐fold change of AKBA‐ versus vehicle‐treated cells. B) Homology model of 15‐LOX‐1 from rabbit 15‐LOX‐1 (blue cartoon, PDB ID: 2P0M) superimposed on AlphaFold 2‐predicted structures from the EMBL databank (red cartoon) and ColabFold predicted structure (white cartoon). Homology model and predicted structures have an RMSD of <1.5 Å for all residues. AKBA (green sticks) docked in interdomain cleft between PLAT domain (blue surface) and catalytic body (white surface). C–E) Three different poses of AKBA (AKBA coordinates extracted from PDB ID: 6NCF) positioned in the interdomain cleft with R98 highlighted from different predicted structures of 15‐LOX‐1. F) LM formation in HEK293 stably transfected with human wt‐15‐LOX‐1 or R98A‐15‐LOX‐1 mutant. Cells (10^6^) were incubated with 1 µm AA, EPA, and DHA (each), and stimulated with AKBA (10 µm) for 180 min at 37 °C. Formed LM were extracted by SPE and analyzed by UPLC/MS‐MS. Data are means ± S.E.M. given as pg/10^6^ cells summarized in groups: 15‐LOX products include 17‐HDHA, 15‐HEPE, and 15‐HETE; 12‐LOX products 14‐HDHA, 12‐HEPE, and 12‐HETE; and 5‐LOX products 5‐HEPE, 5‐HETE, t‐LTB_4_, and LTB_4_; *n* = 3 independent experiments. For statistical analysis, data were log‐transformed, unpaired Student's *t*‐test; **p < 0.01, wt‐15‐LOX‐1 plus treatment versus R98A‐15‐LOX‐1 mutant plus treatment. G) LM‐biosynthetic enzymes that are affected by AKBA.

To further elucidate activation of 15‐LOXs by AKBA in intact cells, we exploited HEK293 cells stably transfected with human 15‐LOX‐1 or 15‐LOX‐2, and cells expressing 5‐LOX or 12‐LOX as controls.^[^ ^11^ ^]^ As reported before, in HEK293 cells expressing 5‐LOX, AKBA stimulated 12‐lipoxygenation of AA and EPA, for example, 12‐HETE formation, due to a shift of 5‐LOX regiospecificity.^[^ ^11^ ^]^ Of interest, AKBA strikingly elevated formation of 17‐HDHA, 15‐HETE, and 15‐HEPE in HEK293 cells expressing 15‐LOX‐1 (≈53‐ to 73‐fold) and 15‐LOX‐2 (≈7‐ to 14‐fold) while 5‐LOX products were minimally increased (**Figure** **5**A and Table S7, Supporting Information). 15‐LOX‐1 is a dual 12/15‐lipoxygenating enzyme while 15‐LOX‐2 is specific for 15‐position.^[^ ^19^ ^]^ In 15‐LOX‐1‐expressing HEK293 cells, 12‐LOX‐related products were formed in a ratio of ≈1:10 as compared to 15‐LOX products, which was not the case for 15‐LOX‐2‐expressing cells (Table S7, Supporting Information). Also, in HEK293 cells expressing 12‐LOX, AKBA was stimulatory and elevated 12‐HETE and 12‐HEPE formation without producing 5‐LOX‐derived LMs (Figure 5A and Table S7, Supporting Information). As expected, in non‐transfected HEK293 cells devoid of LOXs, formation of LM was negligible and AKBA was without stimulatory effects.

AKBA binds to 5‐LOX in a deep groove at the interface of the C2‐like (or Polycystin‐1, LOX, Alpha‐Toxin [PLAT]) and catalytic domains and alters enzyme regiospecificity in an allosteric manner by promoting 12‐lipoxygenation of AA.^[^ ^11^ ^]^ Because a similar interdomain groove is present in 15‐LOXs, AKBA may bind at this site and thus increase 15‐LOX activity. There is no structure of h15‐LOX‐1 in the protein data bank. The closest structure to h15‐LOX‐1 by sequence identity is the rabbit 15‐LOX‐1 structure (PDB ID: 1LOX, 81%^[^ ^20^ ^]^) as well as a subsequent model produced after reanalysis of the diffraction data.^[^ ^21^ ^]^ We included both structures as templates for homology modeling of h15‐LOX‐1 for rigor and reproducibility of the docking results (Figure 5B). Additionally, the protein folding consortium that developed AlphaFold 2 (AF2)^[^ ^22^ ^]^ has a predicted‐structure database^[^ ^23^ ^]^ hosted by EMBL‐EBI. We downloaded the AF2‐predicted structure of h15‐LOX‐1 and used ColabFold to predict five structures of h15‐LOX‐1 without homology model templates. Overall, homology models and predicted structures had an RMSD of <1.5 Å for all residues when compared to one‐another, suggesting that the models converged. Human 15‐LOX‐2 (PDB ID: 4NRE) might also be a likely template for 15‐LOX‐1 since they both oxygenate AA with the same regioselectivity and stereochemistry, but they share only 38% sequence identity. Finally, we chose h5‐LOX as the third template with the identified allosteric site for AKBA^[^ ^11^ ^]^ and a sequence identity of 41% with h15‐LOX‐1.

We utilized AutoDock Vina version 1.1.2 (citation https://doi.org/10.1002/jcc.21334) to determine where AKBA docks to these h15‐LOX‐1 homology models and the AF2‐predicted structures. Docking of AKBA (PDB ligand ID: AF7) was performed using different search volumes, including the entire protein and focused searches in the interdomain cleft between the PLAT and the catalytic domains. Autodock Vina consistently placed AKBA in this interdomain pocket (Figure 5B), populating at least the top three scores with different ligand conformations (Figure 5C–E). This trend was similar for homology‐generated models and AF2‐predicted structures using the different search volumes. Hydrophobic burial of AKBA appears to be the primary molecular determinant for binding. However, in particular, R98 but also H127, R135, and D163 are residues that may participate in H‐bonds and/or electrostatic interactions with carboxyl, *O*‐acetoxy, and/or oxo groups of AKBA (Figure 5C–E).

To test whether AKBA mediates its stimulatory effects via this interdomain pocket, we replaced R98 in h15‐LOX‐1 by Ala to negate the putative favorable interaction with the AKBA carboxy group. The R98A‐15‐LOX‐1 mutant was expressed in HEK293 cells that were stimulated in presence of 1 µm AA, EPA, and DHA, each. The levels of 12/15‐LOX products in the wt‐ and R98A‐expressing cells were similar (without stimulus, Table S8, Supporting Information), but the product levels were significantly increased in the cells expressing wt‐15‐LOX‐1 by AKBA, but not for R98A mutant‐ expressing cells (Figure 5F and Table S8, Supporting Information); wt‐ and R98A‐15‐LOX‐1 enzymes were equally expressed, as determined by Western Blot (Figure S6, Supporting Information). Together, the strong induction of cellular 12/15‐LOX product/SPM formation by AKBA is likely due to direct 15‐LOX activation and translocation to the membrane where newly liberated PUFA are accessed and efficiently converted by the activated 15‐LOX, independent of Ca^2+^ and MAPKs.

### 2.6 AKBA Promotes SPM Formation and Limits PMNL Infiltration in Mouse Peritonitis In Vivo

**Figure 6:**
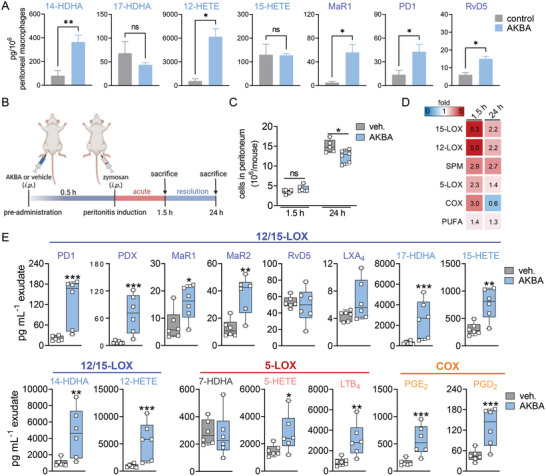
AKBA increases SPM formation in murine peritoneal macrophages and in zymosan‐induced peritonitis. A) Murine peritoneal macrophages were treated with AKBA (10 µm) or vehicle (0.1% DMSO) control for 180 min and released LM were extracted by SPE and analyzed by UPLC‐MS‐MS. Data are means ± S.E.M. given in pg/10^6^ cells, *n* = 4 separate donors. Statistical analysis was done using paired Student's *t*‐test, *p < 0.05, **p < 0.01, AKBA versus vehicle control. B) Experimental timeline of zymosan‐induced murine peritonitis. Male CD1 adult mice (*n* = 6 per group) were treated intraperitoneally (i.p.) with AKBA (20 mg kg^−1^) or vehicle (2% DMSO in 0.9% NaCl) 0.5 h before peritonitis induction with zymosan (1 mg, i.p.). C) Cell numbers in the peritoneal cavity after 1.5 h (acute phase) and 24 h (resolution phase) of peritonitis; *n* = 6 animals each group. Statistical analysis was done using unpaired Student's *t*‐test, *p < 0.05, AKBA versus vehicle control. D) Groups of LMs from peritoneal exudates after 1.5 or 24 h (means of *n* = 6 animals per group) shown as a heat map indicating the ‐fold change of AKBA versus vehicle treatment. 15‐LOX products include 17‐HDHA, 15‐HETE, and 15‐HEPE; 12‐LOX products include 14‐HDHA, 12‐HETE, and 12‐HEPE; SPM include PD1, PDX, RvD5, LXA_4_, MaR1, and MaR2; 5‐LOX products include 5‐HETE, 5‐HEPE, t‐LTB_4_, and LTB_4_; COX products include PGE_2_, PGD_2_, TXB_2_, and PGF_2*α*_; PUFA include AA, EPA, and DHA. E) Box and whiskers blots of selected LM after 1.5 h zymosan‐induced peritonitis in pg mL^−1^ exudate, *n* = 6 animals each group. Data were log‐transformed for statistical analysis, unpaired Student´s *t*‐test, *p < 0.05, **p < 0.01, ***p < 0.001 AKBA versus vehicle.

We investigated if AKBA promotes 12/15‐LOX product/SPM formation in vivo during zymosan‐induced mouse peritonitis, an experimental model of self‐limited acute inflammation controlled by LMs.^[^ ^15^, ^24^ ^]^ Since resident peritoneal macrophages (PM) are major LM‐producing cells in this model, we exposed isolated PMs from mouse peritoneum to AKBA (10 µm) to confirm release of LM. Note that the murine 15‐LOX ortholog mainly oxygenates AA and EPA at C12 and DHA at C14, but less at C15 and C17, respectively.^[^ ^19^ ^]^ AKBA strongly elevated 12‐lipoxygenation (12‐HETE, 14‐HDHA) and SPM formation, especially 14‐HDHA‐derived MaR1 (≈30‐fold) (**Figure** **6**A). Next, mice were treated intraperitoneally (i.p.) with AKBA (20 mg kg^−1^) 0.5 h before zymosan (1 mg, i.p.), and peritoneal exudates were collected 1.5 h (acute phase) and 24 h (resolution phase) post zymosan (Figure 6B). The numbers of infiltrated PMNL in the peritoneal cavity were unchanged after 1.5 h AKBA treatment but after 24 h, cell numbers were significantly reduced (Figure 6C), suggesting enhanced resolution of inflammation. Notably, AKBA‐treatment caused a striking increase of PD1 and PDX and its precursor 17‐HDHA after 1.5 h (≈6.5‐ to 10‐fold), which declined after 24 h (Figure 6D,E). Also, 15‐LOX‐derived MaR1 and MaR2, as well as 14‐HDHA and 12‐HETE, were strongly elevated by AKBA at 1.5 h post zymosan (Figure 6D,E). Formation of 5‐LOX products was also increased by AKBA; the relatively low amounts of COX products (i.e., PGD_2_ and PGE_2_) were elevated by 2.5‐ and 3.7‐fold after 1.5 h but not after 24 h (Figure 6D,E). These elevated 5‐LOX‐ and COX‐derived products were not as pronounced as those formed by 15‐LOX‐1 and might be due to activation of PLA_2_ and thus, elevated PUFA supply (Figure 6D). Together, treatment of mice with AKBA during peritonitis strongly enhances SPM formation, with minor stimulatory effects on pro‐inflammatory PG and LT along with enhanced resolution of inflammation.

## 3 Discussion

Here, we explored our previous discovery of an AKBA‐induced allosteric regiospecificity shift of 5‐LOX from a 5‐ to a 12‐lipoxygenating enzyme.^[^ ^11^ ^]^ In particular and unexpectedly, AKBA evokes activation of cellular 15‐LOX‐1, a key enzyme in SPM biosynthesis,^[^ ^10^ ^]^ resulting in significantly elevated levels of SPMs detected. Compared to classical stimuli like ionophores or bacterial exotoxins, AKBA induces SPM formation by distinct, Ca^2+^‐ and phosphorylation‐independent mechanisms, with only modest induction of 5‐LOX and COX products. Our discovery advances inflammation resolution pharmacology and offers a new concept for treating diseases with unresolved inflammation by using specific allosteric LOX modulators such as AKBA that not only lower the pro‐inflammatory LM but cause a “class‐switch” to the resolution phase through activation of SPMs.

Natural products offer tremendous opportunities for chemical biology and drug discovery, especially to modulate LOX/COX pathways.^[^ ^25^ ^]^ Among the vast number of natural product 5‐LOX inhibitors,^[^ ^25^, ^26^ ^]^ AKBA may represent the most prominent candidate for a therapeutic.^[^ ^27^ ^]^ Numerous clinical trials document the efficacy of AKBA‐containing frankincense preparations in the treatment of rheumatoid arthritis, osteoarthritis, bronchial asthma, inflammatory bowel disease, multiple sclerosis, psoriasis and erythematous eczema, and cancer.^[^ ^27^, ^28^ ^]^ AKBA interferes with multiple pro‐inflammatory target proteins,^[^ ^28^ ^]^ and in addition to 5‐LOX, inhibits COX‐1, microsomal PGE_2_ synthase‐1, and platelet‐type 12‐LOX (Figure 5G);^[^ ^27^ ^]^ all of these are enzymes within the LM network. We found that in line with the shift of 5‐LOX regiospecificity toward a 12/15‐lipoxygenating enzyme,^[^ ^11^ ^]^ AKBA caused a biosynthetic switch from pro‐inflammatory 5‐LOX and COX products to 12/15‐LOX‐derived LM in 5‐LOX‐rich PMNL, monocytes, and macrophages challenged by *E. coli*. These innate immune cells possess high capacities to generate a broad spectrum of LM upon adequate activation.^[^ ^5^, ^6^ ^]^ Among them, macrophages can adopt pro‐inflammatory M1 and pro‐resolving M2 phenotypes^[^ ^29^ ^]^ that are clearly distinguished by their LM signature profiles: M1 mainly produce pro‐inflammatory LTs and PGs, while M2 generate high amounts of SPMs.^[^ ^13^, ^30^ ^]^ In the present study, the highest SPM levels upon *E. coli* challenge were evident in M2‐MDMs as compared to PMNL, monocytes and M1‐MDMs, regardless of AKBA treatment, seemingly due to the strong expression of 15‐LOX‐1 in M2‐MDMs.^[^ ^13^, ^15^ ^]^ Moreover, in M2‐MDM, 5‐LOX was not inhibited by AKBA, enabling elevation of SPMs involving 5‐LOX in their biosynthesis (e.g., LXA_4_, RvD2, and RvD5). 15‐LOX‐1, but not 15‐LOX‐2, is also a 12‐lipoxygenating enzyme,^[^ ^19^ ^]^ which explains the elevated levels of MaR1, 12‐HETE, 12‐HEPE, and 14‐HDHA in AKBA‐treated M2‐MDMs. In monocytes and PMNL, AKBA markedly elevated formation of 12‐LOX products, with minor effects on 15‐lipoxygenated ones, in agreement with the presence of 12‐LOX and absence of 15‐LOXs in these cells.^[^ ^19^ ^]^ Favorable LM class switch features in activated leukocytes were recently also found for the anti‐inflammatory natural product celastrol^[^ ^31^ ^]^ and plant‐derived (dihydro‐)chalcones,^[^ ^32^ ^]^ though the underlying mechanisms remain elusive.

It is intriguing that AKBA not only modulates agonist‐induced LM formation but acts as stimulus for the four leukocyte types to generate LM, suggesting an ability to activate all cellular processes required for LM biosynthesis: 1) release of free PUFA substrates, 2) subcellular redistribution of LOX to access liberated PUFA, and 3) LOX activation to oxygenate PUFA.^[^ ^6^, ^17^, ^19^ ^]^ Notably, however, the LM profiles and the underlying mechanisms evoked by AKBA differed clearly from other agonists. Thus, ionophore rapidly induced mainly pro‐inflammatory 5‐LOX products in MDMs, as observed by others,^[^ ^33^ ^]^ with modest elevations by AKBA that in contrast, evoked mainly 12/15‐LOX products/SPMs in a delayed manner, especially in M2‐MDMs. The latter supports that AKBA acts via 15‐LOX‐1 that is most abundantly expressed in M2‐MDMs versus the other three cell types,^[^ ^13^, ^19^ ^]^ much more than the 15‐LOX‐2 isoform;^[^ ^15^ ^]^ in fact, silencing 15‐LOX‐1 in M2‐MDMs clearly reduced AKBA‐evoked 12/15‐LOX product formation.

In general, agonists like ionophore A23187 and bacterial exotoxins activate PLA_2_ and 5‐/15‐LOX through elevation of [Ca^2+^]_I_ and/or phosphorylation by MAPK via subcellular redistribution of the enzymes.^[^ ^13^, ^16^, ^18^, ^34^ ^]^ AKBA increased [Ca^2+^]_I_ and activated p38 MAPK and ERK‐1/2 in human PMNL and elevated [Ca^2+^]_I_ in MDMs.^[^ ^14^, ^27^ ^]^ However, in contrast to ionophore A23187 that massively and rapidly elevates [Ca^2+^]_I_ along with strong 5‐LOX activation,^[^ ^18^ ^]^ AKBA‐induced 15‐LOX‐1 translocation and product formation in M2‐MDMs were Ca^2+^‐independent and delayed. Note that 5‐LOX and 15‐LOX‐1 translocate to different compartments upon stimulation with AKBA where distinct PLA_2_ isoforms may reside that supply PUFAs to the two LOXs. Such distinct and temporal activation of 5‐LOX and 15‐LOX‐1 was shown also for stimulation with *Staphylococcus aureus*‐derived *α*‐hemolysin, where a delayed Ca^2+^ influx in M2‐MDMs activated 15‐LOX‐1 compared to the rapid and strong Ca^2+^ influx obtained after A23187 treatment stimulating 5‐LOX.^[^ ^15^ ^]^ LOX activation via phosphorylation^[^ ^16^, ^18^ ^]^ is excluded, as AKBA failed to stimulate relevant kinases in MDMs, and respective kinase inhibitors did not block AKBA‐induced LOX product formation.

Liberation of PUFA by PLA_2_ enzymes is a prerequisite for agonist‐induced LM formation,^[^ ^6b^ ^]^ and AKBA promoted AA release in human PMNL and platelets connected to eicosanoid generation.^[^ ^14^, ^27^ ^]^ Also in MDMs, AKBA strongly elevated AA, EPA, and DHA levels, which may account for the marked 12/15‐LOX product/SPM generation. AKBA may activate cPLA_2_ for PUFA release, as this PLA_2_ is commonly involved in LM formation in leukocytes and, like 5‐LOX, possesses a PLAT domain that is required to access phospholipid substrates;^[^ ^16b^ ^]^ whether AKBA does bind and activate cPLA_2_ via its PLAT domain remains to be investigated. However, AKBA was still able to increase LM formation when endogenous substrate supply was circumvented in M2‐MDMs and HEK293 cells by exogenous PUFA provision, supporting additional 15‐LOX‐1‐activating features with some minor effects on 15‐LOX‐2 and p12‐LOX.

The 15‐LOX‐stimulatory functions of AKBA strongly depended on its structure, as BAs without the 11‐keto moiety were inactive, and KBA, lacking the 3‐*O*‐acetly group, was less active. Similar SARs were evident for interference with 5‐LOX, COX‐1, and p12‐LOX, where among the four natural *β*BAs from frankincense, AKBA was most potent.^[^ ^14^, ^27^ ^]^ In 5‐LOX, AKBA binds in a deep groove between the PLAT and catalytic domains distant from the enzyme's active site to promote 12/15‐lipoxygenation of AA.^[^ ^11^ ^]^ We observed a similar interdomain groove present in homology models and AF2‐predicted structures of 15‐LOXs. AKBA was reproducibly docked in this interdomain groove previously identified for 5‐LOX (PDB ID: 6NCF) with the 3‐*O*‐acetly group or carboxyl group interacting with the positively charged R98. Replacement of R98 by alanine resulted in impaired activation of this 15‐LOX mutant in HEK293 cells by AKBA compared to the wild‐type enzyme, supporting the idea that the interdomain cleft is an allosteric site for AKBA. Recently, 15‐LOX activators were suggested as anti‐inflammatory therapeutics^[^ ^35^ ^]^ that, based on molecular dynamics simulations,^[^ ^36^ ^]^ were proposed to shift the AA metabolic network toward inflammation resolution by allosteric 15‐LOX activation at a hypothetical second AA binding site. Experimental data confirming such shift in cells or in vivo are missing.

Our finding that AKBA shifts LM formation toward SPMs as immunoresolvents that enhance resolution of the inflammatory process might be exploited as novel pharmacological strategy for intervention with inflammation‐driven diseases. SPMs limit excessive PMNL tissue infiltration, stimulate local immune cell (e.g., macrophage)‐mediated clearance of apoptotic PMNL and cellular debris, and counter‐regulate pro‐inflammatory eicosanoid/cytokine release.^[^ ^5b^ ^]^ Current standard anti‐inflammatory therapeutics are NSAIDs, steroids, disease‐modifying antirheumatic drugs, and biologicals that dampen symptoms of inflammation but exert severe side effects and can derail pro‐resolution pathways, and thus, propagate the underlying disease.^[^ ^37^ ^]^ In contrast, resolution‐based therapeutic strategies using SPM are promising, new approaches for treatment of inflammatory pathologies by reverting the disease with tissue‐protective mechanisms.^[^ ^7^, ^37^, ^38^ ^]^ In particular, SPM‐based therapies may foster inflammation resolution less burdened by unwanted effects than existing therapies with chronic use of NSAIDs or steroids, for example, in arthritis patients.^[^ ^37^ ^]^ In this respect, an AKBA‐enriched frankincense extract conferred statistically significant improvements in pain and physical function scores and unchanged safety parameters in a comprehensive trial with osteoarthritis patients versus placebo.^[^ ^39^ ^]^ Finally, inflammation is a hallmark of cancer^[^ ^3^ ^]^ and stimulation of inflammation resolution by SPM can prevent carcinogenesis by clearance of cellular debris via macrophage efferocytosis and inhibition of pro‐inflammatory eicosanoid/cytokine storms.^[^ ^40^ ^]^ Frankincense preparations suppressed proliferation, angiogenesis, invasion, and metastasis in various cancer types and thus possess clinical potential for cancer therapy.^[^ ^28^ ^]^ Accordingly, elevation of SPM by AKBA during therapy with frankincense might be beneficial for host‐targeted cancer therapy.

In addition to increasing EPA/DHA intake for enhancing endogenous SPM levels,^[^ ^7^ ^]^ therapeutic SPM application is currently being evaluated in clinical trials for treatment of eye inflammation and periodontal diseases.^[^ ^41^ ^]^ Alternative SPM‐based strategies comprise elevation of endogenous SPM levels by smart pharmacological approaches,^[^ ^13^, ^25^ ^]^ Thus, anabasum, a synthetic analog of Δ9‐trans‐tetrahydrocannabinol, was shown to lower LT and PG but elevate SPM levels in acute inflammation driven by UV‐killed *E. coli* in healthy humans along with enhanced resolution.^[^ ^42^ ^]^ AKBA or frankincense preparations could also be promising candidates for such strategies. Although 15‐LOX activation by AKBA remains to be confirmed in vivo, our results from the zymosan‐induced peritonitis, an experimental mouse model of self‐limited acute inflammation,^[^ ^15^, ^24^ ^]^ are supportive in this respect. Thus, AKBA‐treatment strikingly increased PDs and MaRs and their 15‐LOX‐derived precursor 17‐HDHA and 14‐HDHA with minor stimulatory effects on PG and LT, culminating in enhanced inflammation resolution mirrored by impeded PMNL infiltration. Future mechanistic studies in vivo using animal models of inflammation may reveal the proof‐of‐concept and pharmacological relevance of allosteric AKBA‐induced 15‐LOX activation and SPM formation.

## 4 Conclusion

Effective inflammation resolution programs are key for avoiding the progression to excessive and chronic inflammatory diseases and may be accomplished by SPM as potent immunoresolvents that revert the disease with tissue restoring mechanisms. Along these lines, our discovery of AKBA as a molecular switch enabling innate immune cells to block formation of pro‐inflammatory eicosanoids and generate SPM by modulation of 5‐ and 15‐LOX isoforms at allosteric sites, may give direction for development of new SPM‐based pro‐resolving therapeutics.

## 5 Experimental Section

### Isolation and Culture of Human Cells

PMNL and monocytes were isolated from leukocyte concentrates that were obtained from freshly withdrawn peripheral blood of healthy adult male and female donors with informed written consent at the Institute of Transfusion Medicine, University Hospital Jena, Germany. The experimental protocols were approved by the ethical committee of the University Hospital Jena (approval no. 5050‐01/17) and all experimental procedures were performed in accordance with the relevant guidelines and regulations. For sedimentation of erythrocytes, the leukocyte concentrates were mixed with dextran from *Leuconostoc* spp. (MW ≈ 40 000, Sigma Aldrich). The supernatants were centrifuged on lymphocyte separation medium (Histopaque‐1077, Sigma Aldrich). Contaminating erythrocytes in the sedimented PMNL were removed by hypotonic lysis using distilled water, and PMNL were washed two times with ice‐cold PBS pH 7.4 and then resuspended in PBS pH 7.4 plus 1 mg mL^−1^ glucose (PG buffer). The peripheral blood mononuclear cell fraction was seeded in RPMI 1640 (Sigma‐Aldrich), supplemented with 10% v/v heat‐inactivated fetal calf serum (FCS), 100 U mL^−1^ penicillin, and 100 µg mL^−1^ streptomycin in tissue culture flasks (Greiner Bio‐one, Frickenhausen, Germany) and kept for 1.5 h at 37 °C and 5% CO_2_ to adhere monocytes.

To differentiate monocytes to macrophages and further polarization toward M1‐ and M2‐MDMs, published criteria and procedures were applied.^[^ ^13^, ^29^ ^]^ Briefly, M1‐MDMs were obtained by differentiating monocytes with 20 ng mL^−1^ GM‐CSF (Peprotech, Hamburg, Germany) for 6 days in RPMI 1640 supplemented with 10% FCS, 2 mmol L^−1^ L‐glutamine (Biochrom/Merck, Berlin, Germany), and penicillin–streptomycin (Biochrom/Merck), followed by incubation with 20 ng mL^−1^ IFN‐*γ* (Peprotech) and 100 ng mL^−1^ LPS for another 48 h. M2‐MDMs were obtained by differentiating monocytes with 20 ng mL^−1^ M‐CSF (Peprotech) for 6 days and subsequent polarization using 20 ng mL^−1^ IL‐4 (Peprotech) for 48 h.

HEK293 cells stably expressing FLAP and 5‐LOX were selected using 200 µg mL^−1^ hygromycin B and 400 µg mL^−1^ geneticin, as described before.^[^ ^43^ ^]^ HEK293 cells expressing 12‐LOX, 15‐LOX‐1, or 15‐LOX‐2 using the plasmids pcDNA3.1/neom (+)_12‐LOX, pCMV6_15‐LOX‐1 (Origene, NM_001140) or pcDNA3.1/neom (+) 15‐LOX‐2 were selected using 400 µg mL^−1^ geneticin as reported previously.^[^ ^11^ ^]^

### Side‐Directed Mutagenesis and Stable Expression of LOXs in HEK293 Cells

The pCMV6‐AC_15‐LOX‐1 vector was used as a template for amplification. The mutation was introduced with the Q5 Site‐Directed Mutagenesis kit according to the manufacturer's (New England Biolabs) instructions. Oligonucleotides of 20–30 bases were ordered from Eurofins genomics (forward 5′‐cccttgttacgcctgggtggagggc, reverse 5′‐aacctgacctcgtccccg). The mutated vector was transformed in chemically competent Top10 *E. coli* (Invitrogen) and isolated using GeneJET Plasmid‐Miniprep‐kit according to the manufacturer's (Thermo Scientific) instructions. The mutation was confirmed by sequencing using a 15‐LOX‐1 specific customized oligonucleotide (5′‐gccgctgctgtttgtgaaactg, Eurofins Genomics).

Transfection of HEK293 cells with pCMV5‐AC_15‐LOX‐1 mutants was performed as described before.^[^ ^43^ ^]^ HEK293 cells were transfected using the Lipofectamine LTX with Plus Reagent according to the manufacturer's (Invitrogen) instructions. In brief, cells were cultured until ≈60% confluency and 1 day prior to transfection the medium was replaced by culture medium devoid of antibiotics. Transfection mix (6.25 µg purified vector) was added dropwise. The medium was replaced after 24 h by culture medium containing antibiotics and incubated for another 24 h, before 15‐LOX‐1‐expressing cells were selected by 400 µg mL^−1^ geneticin. Sable transfectants were verified by immunoblotting and assayed for 15‐LOX‐1 activity.

### Cell Viability and Cell Integrity Analysis Using MTT Assay and LDH Release Assay

M1‐ or M2‐MDMs (10^5^ mL^−1^) were treated in a 96‐well plate in RPMI 1640 supplemented with 10% v/v FCS, 100 U mL^−1^ penicillin, and 100 µg mL^−1^ streptomycin with BAs or 0.1% vehicle (DMSO) for 180 min; 1% triton X‐100 was used as positive control.

For analysis of cell viability, cells were incubated with MTT (5 mg mL^−1^, 20 µL; Sigma‐Aldrich, Munich, Germany) for 2–3 h at 37 °C and 5% CO_2_ in the darkness. The formazan product was solubilized with sodium dodecyl sulfate (SDS, 10% in 20 mm HCl) and the absorbance was monitored at 570 nm using a Multiskan Spectrum microplate reader (Thermo Fisher Scientific, Schwerte, Germany).

For analysis of cell integrity, the release of LDH was assessed by CytoTox 96 Non‐Radioactive Cytotoxicity assay according to the manufacturer´s (Promega, Mannheim, Germany) instructions. Cells were centrifuged at 400 × *g* (5 min, 4 °C) and the supernatants were diluted to appropriate LDH concentrations. The absorbance was monitored at 490 nm using a NOVOstar microplate reader (BMG Labtechnologies GmbH, Offenburg, Germany). Cell integrity was calculated according to the manufacture´s guidelines.

### Cell Incubations and LM Metabololipidomics by UPLC‐MS‐MS

Freshly isolated PMNL and monocytes as well as polarized MDMs and HEK293 cells (1 or 2 × 10^6^ in 1 mL, as indicated) were incubated in PG buffer containing 1 mm CaCl_2_. In some experiments, cells were incubated in PG buffer containing 0.5 mm EDTA or 0.5 mm EDTA plus BAPTA/AM (20 µm). AKBA and other compounds, *E. coli* (O6:K2:H1; ratio 1:50) or vehicle (0.1% DMSO) as well as AA, EPA, and DHA were added at 37 °C as indicated. After the indicated (pre‐)incubation times, the supernatants (1 mL) were mixed with 2 mL of ice‐cold methanol that contained deuterium‐labeled internal standards (200 nm d4‐PGE_2_, d4‐LTB_4_, d5‐LXA_4_, d5‐RvD2, and d8‐5S‐HETE, as well as 10 µm d8‐AA; Cayman Chemical/Biomol GmbH, Hamburg, Germany). Solid phase extraction (SPE) of LM, sample preparation, and UPLC‐MS‐MS analysis of LM was performed as described by Jordan et al.^[^ ^15^ ^]^

### ALOX15A Gene Knockdown in Human M2‐MDMs

Freshly isolated monocytes were differentiated with M‐CSF (20 ng mL^−1^) to MDM_M‐CSF_ and then subjected to electroporation using Neon Transfection System 100 µL Kit (Thermo Fisher Scientific) as described by Jordan et al.^[^ ^15^ ^]^ In brief, MDM_M‐CSF_ (1.5 × 10^6^) were resuspended in 1 mL of PBS and 20 µm of human non‐targeting siRNA (Thermo Fisher Scientific; D‐001810‐01‐05) or 20 µm of ALOX15A Trilencer‐27 Human siRNA (OriGene, SR300171) were added to the cells prior to electroporation. The electroporated cells were resuspended in 1 mL of the corresponding growth medium in the presence of 20 ng mL^−1^ IL‐4 (Peprotech) and incubated for 48 h at 37 °C and 5% CO_2_.

### SDS‐PAGE and Western Blot

Cell lysates corresponding to 2 × 10^6^ M2‐MDM were separated on 10% polyacrylamide gels and blotted onto nitrocellulose membranes (Amersham Protran Supported 0.45 µm nitrocellulose, GE Healthcare, Freiburg, Germany). The following primary antibodies were used for protein detection: rabbit polyclonal anti‐5‐LOX, 1:1000 (Genscript, Piscataway to a peptide with the C‐terminal 12 amino acids of 5‐LOX: CSPDRIPNSVAI); mouse monoclonal anti‐15‐LOX‐1, 1:500 (ab119774, Abcam, Cambridge, UK); rabbit polyclonal anti‐15‐LOX‐2, 1:500 (ab23691, Abcam); mouse monoclonal anti‐phospho‐p44/42 MAPK (ERK‐1/2) (Thr202/Tyr204), 1:750 (#9106, Cell Signaling); rabbit monoclonal anti‐p44/42 MAPK (ERK‐1/2), 1:1000 (#4695, Cell Signaling); rabbit polyclonal anti‐phospho‐p38 MAPK (Thr180/Tyr182), 1:750 (#9211, Cell Signaling); rabbit monoclonal anti‐p38 MAPK, 1:1000 (D13E1, #8690, Cell Signaling); rabbit polyclonal anti‐phospho‐SAPK/JNK (Thr183/Tyr185), 1:750 (#9251, Cell Signaling); rabbit polyclonal anti‐SAPK/JNK, 1:1000 (#9252, Cell Signaling); rabbit polyclonal anti‐phospho‐Akt (Ser473), 1:750 (#9271, Cell Signaling); mouse monoclonal anti‐Akt, 1:1000 (40D4, #2920, Cell Signaling); rabbit monoclonal anti‐GAPDH, 1:1000 (D16H11, Cell Signaling); and mouse monoclonal anti‐*β*‐actin, 1:1000 (8H10D10, Cell Signaling). Immunoreactive bands were stained with IRDye 800CW Goat anti‐Mouse IgG (H+L), 1:10 000 (926‐32210, LI‐COR Biosciences, Lincoln, NE), IRDye 800CW Goat anti‐Rabbit IgG (H+L), 1:15 000 (926 32211, LI‐COR Biosciences) and/or IRDye 680LT Goat anti‐Mouse IgG (H+L), 1:40 000 (926‐68020, LI‐COR Biosciences), and visualized by an Odyssey infrared imager (LI‐COR Biosciences, Lincoln, NE). The data from densitometric analyses were background corrected.

### [^3^H]‐Arachidonic Acid Labeling of Macrophages and Measurement of AA Metabolites

The release of [^3^H]‐AA and its transformed metabolites from macrophages was analyzed as described before.^[^ ^44^ ^]^ Briefly, cells were resuspended in RPMI 1640 medium supplemented with 10% v/v FCS, 100 U mL^−1^ penicillin, and 100 µg mL^−1^ streptomycin and incubated with 5 nm tritium‐labeled [5,6,8,9,11,12,14,15‐^3^H]AA (Biotrend Chemicals GmbH, Cologne, Germany) for 24 h at 37 °C. Cells were washed and resuspended in PG buffer containing 1 mm CaCl_2_, incubated with 10 µm RSC3388 for 15 min, and then stimulated with 0.5 µm A23187 or 10 µm AKBA for 90 min at 37 °C. The reaction was stopped on ice, cells were centrifuged and aliquots (800 µL) of the supernatants were combined with 3 mL Rotiszint eco plus and assessed for radioactivity by scintillation counting on a Micro Beta Trilux (Perkin Elmer, Waltham, MA) as reported.^[^ ^44^ ^]^

### Immunofluorescence Microscopy

Analysis of the subcellular localization of 5‐LOX and 15‐LOX‐1 in M2‐MDM by immunofluorescence microscopy was performed as described by Jordan et al.^[^ ^15^ ^]^ In brief, M2‐MDMs (10^6^ cells) were cultured on glass coverslips for 48 h and the medium was then replaced by PG buffer containing 1 mm CaCl_2_. In some experiments, cells were incubated in PG buffer containing 0.5 mm EDTA or 0.5 mm EDTA plus BAPTA/AM (20 µm). BAs, A23187, or vehicle (0.1% DMSO) were added, and incubations were kept at 37 °C for the indicated times. Cells were then fixed, permeabilized, blocked with normal goat serum (10%, 50062Z, Thermo Fisher Scientific), and then incubated with rabbit anti‐5‐LOX antibody, 1:100 (1550 AK6, kindly provided by Dr. Olof Radmark, Karolinska Institutet, Stockholm, Sweden) or mouse monoclonal anti‐15‐LOX‐1 antibody, 1:100 (ab119774, Abcam, Cambridge, UK) at 4 °C overnight. Staining of 5‐LOX and 15‐LOX‐1 with fluorophore‐labeled secondary antibodies and sample analysis using a Zeiss Axiovert 200 M microscope and a Plan Neofluar 40×/1.30 Oil (DIC III) objective (Carl Zeiss, Jena, Germany) was performed as described.^[^ ^15^ ^]^

### Ca^2+^ Imaging

Analysis of intracellular Ca^2+^ levels in M1‐ and M2‐MDMs was performed as described by Jordan et al.^[^ ^15^ ^]^ Briefly, M1‐ and M2‐MDMs were pre‐stained with Fura‐2AM (1 µm) for 30 min at 37 °C and then resuspended in Krebs–Hepes buffer containing 0.1% BSA (1.25 × 10^6^ cells per mL) and 1 mm CaCl_2_ was added. After 10 min, 2 µm ionomycin, 10 µm BAs, or vehicle (0.1% DMSO) were added and the signal was monitored in a thermally (37 °C) controlled NOVOstar microplate reader (BMG Labtechnologies GmbH). Emission at 510 nm and excitation at 340 nm (Ca^2+^‐bound Fura‐2) and 380 nm (free Fura‐2) were recorded. Cell lysis with triton X‐100 gave maximal fluorescence signals (= 100%) and chelation of Ca^2+^ with 20 mm EDTA gave minimal fluorescence signals (= 0%).

### Animals

Male CD‐1 mice (33–39 g, 8–9 weeks, Charles River Laboratories, Calco, Italy) were kept in a defined environment (21 ± 2 °C) and provided with standard rodent chow and water. The animals were allowed to acclimate for 4 days before experiments were started and were subjected to a 12 h light/12 h dark schedule; experiments were conducted during the light phase. Animals were randomly assigned for experiments. The experimental procedures were approved by the Italian Ministry according to International and National law and policies (EU Directive 2010/63/EU and Italian DL 26/2014 for animal experiments, ARRIVE guidelines and the Basel declaration including the 3R concept).

Resident PMs were obtained after lavage of the peritoneal cavity of the mice with 7 mL of cold Dulbecco's Modified Eagle's Medium containing 5 U mL^−1^ heparin. PMs were centrifuged (500 × *g* at 4 °C, 5 min) and incubated for 24 h at 37 °C prior to treatment. Cells (2 × 10^6^) were then treated for 90 min at 37 °C as indicated. The samples were centrifuged (500 × *g*, 4 °C, 5 min), supernatants were collected, frozen (−80 °C), and formed LMs were extracted by SPE and then analyzed by UPLC‐MS‐MS as described above.

### Zymosan‐Induced Peritonitis in Mice

Peritonitis in male mice was induced as described before.^[^ ^31^, ^45^ ^]^ In brief, mice (*n* = 6/group) received i.p. 20 mg kg^−1^ AKBA or vehicle (2% DMSO in saline) in 0.5 mL saline per mouse. After 30 min, peritonitis was induced by injection of zymosan (1 mg per mouse in 0.5 mL saline, i.p.). After 1.5 and 24 h, the mice were euthanized using a saturated CO_2_ atmosphere and peritoneal exudates were collected. Thus, the peritoneal cavity was washed with 3 mL ice‐cold PBS and the exudates were subsequently centrifuged at 18 000 × *g* for 5 min at 4 °C. The pelleted cells were counted in a Burker's chamber after vital trypan blue staining by using a light microscope. Cell‐free supernatants of the exudates were immediately frozen and stored at −80 °C for LM analysis by UPLC‐MS/MS as described above.

### Generation of Models to Human 15‐LOX‐1

Homology models of human 15‐LOX‐1 were created through the SWISS‐MODEL web server using templates of rabbit 15‐LOX‐1 (1lox.pdb) and the reanalysis of rabbit 15‐LOX‐1 (2p0m.pdb) and human 5‐LOX (3o8y.pdb).^[^ ^20^, ^21^, ^46^ ^]^ Rabbit 15‐LOX‐1 shares 81% sequence identity with h15‐LOX‐1 and scores as the best template for generating the homology model. Although porcine 12‐LOX (3rde.pdb) shares 86% sequence identity with h15‐LOX‐1, the structure lacks the *β*‐barrel membrane‐binding PLAT (Polycystin‐1, LOX, and Alpha Toxin) domain.)^[^ ^47^ ^]^ Since AKBA had shown to bind in a crevice between the PLAT and catalytic domain of human 5‐LOX (6ncf.pdb), models which were incomplete were not suitable for docking trials.^[^ ^11^ ^]^ The human 12‐LOX structure (3d3l.pdb) determined by Critical Assessment of protein Structure Prediction community was also lacking key peptide regions, rendering it a poor model for docking. Surprisingly, human 15‐LOX‐2 was a poorer template model for h15‐LOX‐1 due to only sharing 38% sequence identity as compared to 41% for h5‐LOX, even though h15‐LOX‐1 and h15‐LOX‐2 both generate the 15*S*‐isomer of HPETE from AA. h5‐LOX was selected as a third template for homology modeling due to the already confirmed allosteric site for pentacyclic triterpenoids and the second highest sequence identity for a complete structure. The AlphaFold 2 predicted structure of human 15‐LOX‐1 was downloaded from the EMBL‐EBI. An advanced notebook of ColabFold was used to predict five models of human 15‐LOX‐1 without the use of homology templates. The MSA coverage was consistently above 1500 sequences for all positions when using the mmseqs2 method offered through ColabFold,^[^ ^48^ ^]^ and the pLDDT confidence values consistently scored >93.0 suggesting a very high confidence of the predicted structure. All models were prepared for docking by adding hydrogens and assigning charges to the macromolecule with Amber. Since models were generated by assigning side‐chain orientations to the most often populated state, the rotamers of K126, E130, and E134 were switched to the third most populated states to allow for more accessibility to the interdomain cleft. These solvent exposed side‐chains should be highly dynamic and explore alternate conformations in the ps time range.^[^ ^49^ ^]^

### Docking of AKBA into Homology Models of Human 15‐LOX‐1

Docking was performed to models of human 15‐LOX‐1 from templates of rabbit 15‐LOX‐1 (1lox.pdb), the reanalysis of rabbit 15‐LOX‐1 (2p0m.pdb), and human 5‐LOX (3o8y.pdb) and AF2‐predicted structures. AutoDock Vina version 1.1.2 (https://doi.org/10.1002/jcc.21334) was utilized via UCSF Chimera for analysis and visualization of the docking of AKBA. A focused approach of rotamer switching was applied to residues in the interdomain cleft to improve ligand accessibility. Docking was performed in each homology model and predicted structures utilizing different target search volumes, which included the entire protein as well as volumes surrounding the cleft between the PLAT and catalytic domains. More specifically, the grid that focused on the interdomain cleft encapsulated the N‐terminal region of the protein in an ≈40 × ≈60 × ≈50 Å box with the cleft positioned roughly in the center. The grid was more than double in volume when all the accessible surface of the protein was included (with dimensions ≈90 × ≈60 × ≈75 Å. Regardless of the volume searched, docking scores were consistently two units better for AKBA to models in which the cleft was made more accessible and Autodock Vina consistently placed AKBA in the interdomain allosteric site corresponding to the recently described site for AKBA with h5‐LOX.^[^ ^11^ ^]^ In docks where the models had the narrowed cleft due to side‐chains blocking the fully opened vestibule, AKBA was still placed in this similar region but with less hydrophobic burial of the triterpenoid.

### Statistics

The results were expressed as means ± standard error of the mean of *n* measurements, where *n* represented the number of experiments with separate donors, performed on different days, as indicated in the legends to the figures. For the in vivo studies, *n* represented the number of animals. Neither was the number of experiments pre‐determined by statistical methods, nor was samples blinded or the data confirmed as normally distributed. GraphPad Prism 8 software (San Diego, CA) was used for data analysis. Two‐tailed *t*‐test was applied for comparison of two groups while for multiple comparison, one‐way and two‐way analysis of variance with Dunnett´s or Tukey's or post hoc tests were used, as indicated. The criterion for statistical significance was *p* < 0.05.

## Conflict of Interest

The authors declare no conflict of interest.

## Author Contributions

J.G. and O.W. designed the study. F.B. performed cell incubations and LM analysis, immunofluorescence microscopy studies, and analysis of intracellular Ca^2+^ levels. S.P. and A.R. performed animal studies and evaluated cell infiltration and performed LM analysis of murine samples. P.M.J. analyzed data and prepared the graphs and figures. M.G. provided materials and gave intellectual advice. N.C.G. and M.E.N. generated homology models of human 15‐LOX‐1 and performed the docking simulations. F.B., P.M.J., N.C.G., M.E.N., and O.W. wrote the manuscript and all authors contributed to data interpretation and manuscript preparation.

## Supporting information

Supporting Information

## Data Availability

The data that support the findings of this study are available from the corresponding author upon reasonable request.
