# Supplementary material for: Allosteric Activation of 15‐Lipoxygenase‐1 by Boswellic Acid Induces the Lipid Mediator Class Switch to Promote Resolution of Inflammation
Source: Adv Sci (Weinh). 2022 Dec 25;10(6):2205604. doi: 10.1002/advs.202205604 (PMC9951388; doi:10.1002/advs.202205604)
Supplement: Supplementary file 1 — Supporting Information [file ADVS-10-2205604-s001.pdf]

Supporting Information

Allosteric activation of 15-lipoxygenase-1 by boswellic acid induces the lipid mediator class switch to promote resolution of inflammation

*Friedemann Börner, Simona Pace, Paul M. Jordan, Jana Gerstmeier, Mario Gomez, Antonietta Rossi, Nathaniel C. Gilbert, Marcia E. Newcomer, Oliver Werz**

Contents:

Figure S1. Effects of AKBA on cell viability of human M2-MDMs.

Figure S2. Effects of boswellic acids on the cell viability, AA release, and 15-LOX-1 subcellular distribution in human MDMs.

Figure S3. Protein expression of 5-LOX and 15-LOX-2 in ALOX15A siRNA-treated M2-MDMs.

Figure S4. Effects of boswellic acids on $[Ca^{2+}]i$ in M1-MDMs.

Figure S5. Effect of AKBA on protein kinase activation and on LM-biosynthetic enzyme expression.

Figure S6. Expression of wt- and R98A-mutant-15-LOX-1 in HEK293 cells at the protein level.

Table S1. Modulation of lipid mediator formation in activated 5-LOX-expressing immune cells by AKBA.

Table S2. Induction of lipid mediator formation in unstimulated 5-LOX-expressing immune cells by AKBA.

Table S3. Effect of AKBA after 15-LOX-1 knockdown in M2 macrophages

Table S4. Effect of AKBA on M2-MDMs after DHA/EPA supplementation.

Table S5. 15-LOX-1 activation by AKBA in M2-MDMs is insensitive to Ca^{2+}.

Table S6. Effects of kinase inhibitors on AKBA-induced LM formation in M2-MDM.

Table S7. Effects of AKBA on LM formation in LOX-transfected HEK293 cells.

Table S8. Effects of AKBA on mutated 15-LOX-1 (R98A) in stable transfected HEK293 cells.

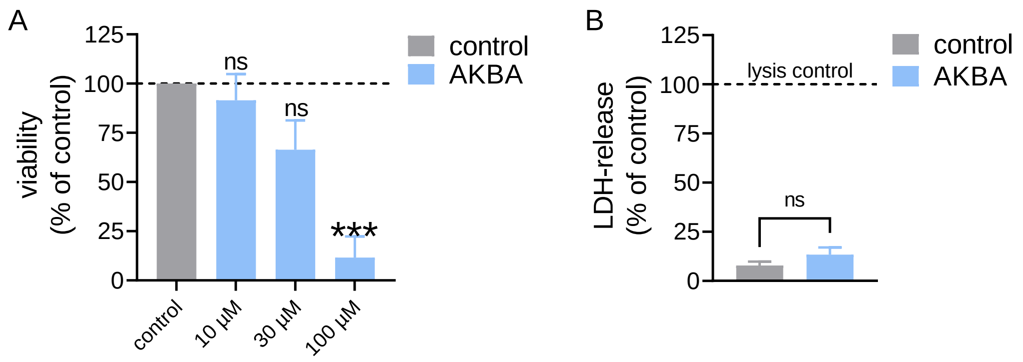

Figure S1. Effects of AKBA on cell viability of human M2-MDMs. (A) MTT cell viability assay of M2-MDMs. 10^5 cells were polarized for 48 h and treaded with AKBA (as indicated) or vehicle (0.1% DMSO) for another 3 h at 37°C (5% CO_2). After addition of MTT solution, cells were incubated for 2 h at 37°C and lysed in an SDS-containing buffer (10% *w/v*). Cell viability is represented by reduction of MTT and shown as percentage of vehicle control. For statistical analysis data was log transformed, unpaired Student's t-test, ***p < 0.001 for AKBA vs. control, n = 4 separate donors. (B) LDH release of M2-like MDMs after exposure to AKBA (10 µM) or vehicle. Triton X-100 was used as a positive control (max. LDH release) and percent cytotoxicity were calculated referred to manufacturer´s guidelines; unpaired Student's t-test, n = 3 separate donors.

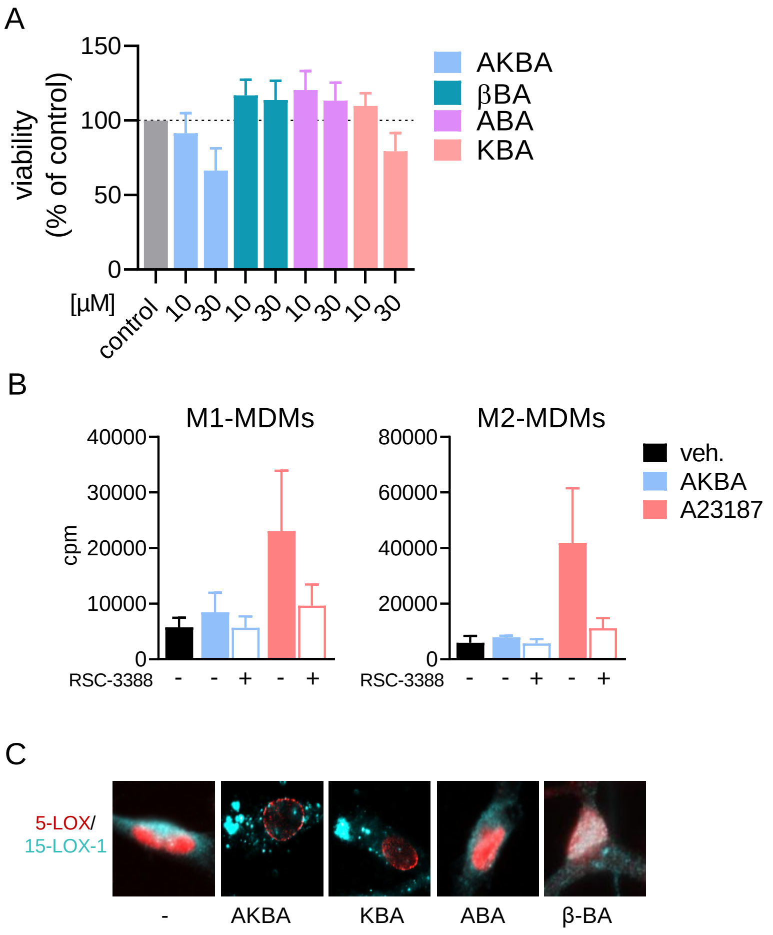

Figure S2. Effects of boswellic acids on the cell viability, AA release, and 15-LOX-1 subcellular distribution in human MDMs. (A) MTT cytotoxicity assay of human M2-like MDMs. 10^5 cells were treated with BAs (as indicated) or vehicle (0.1% DMSO) for 180 min at 37 °C. After addition of MTT solution cells were incubated for another 120 min at 37°C and lysed in a SDS containing buffer (10% *w/v*). Cell viability is shown as percentage of vehicle control; n = 3 - 4 separate donors. (B) Release of [^{3}H]-AA and its metabolite from [^{3}H]-AA-prelabelled M1- and M2-MDM preincubated with 10 µM RSC-3388 for 10 min and stimulated with A23187 (0.5 µM) or AKBA (10) for 10 and 90 min, each; results are given in cpm (counts per minute), means + S.E.M.; n = 3. (C) Subcellular redistribution of 5-LOX and 15-LOX-1 in M2-MDMs after exposure to with boswellic acids (BA, 10 µM each) for 180 min. Cells were fixed, permeabilized, and incubated with antibodies against 5-LOX (red) and 15-LOX-1 (cyan-blue); scale bars = 10 µm. Results shown for one single cell are representative for approx. 100 individual cells analysed, n = 3 independent experiments.

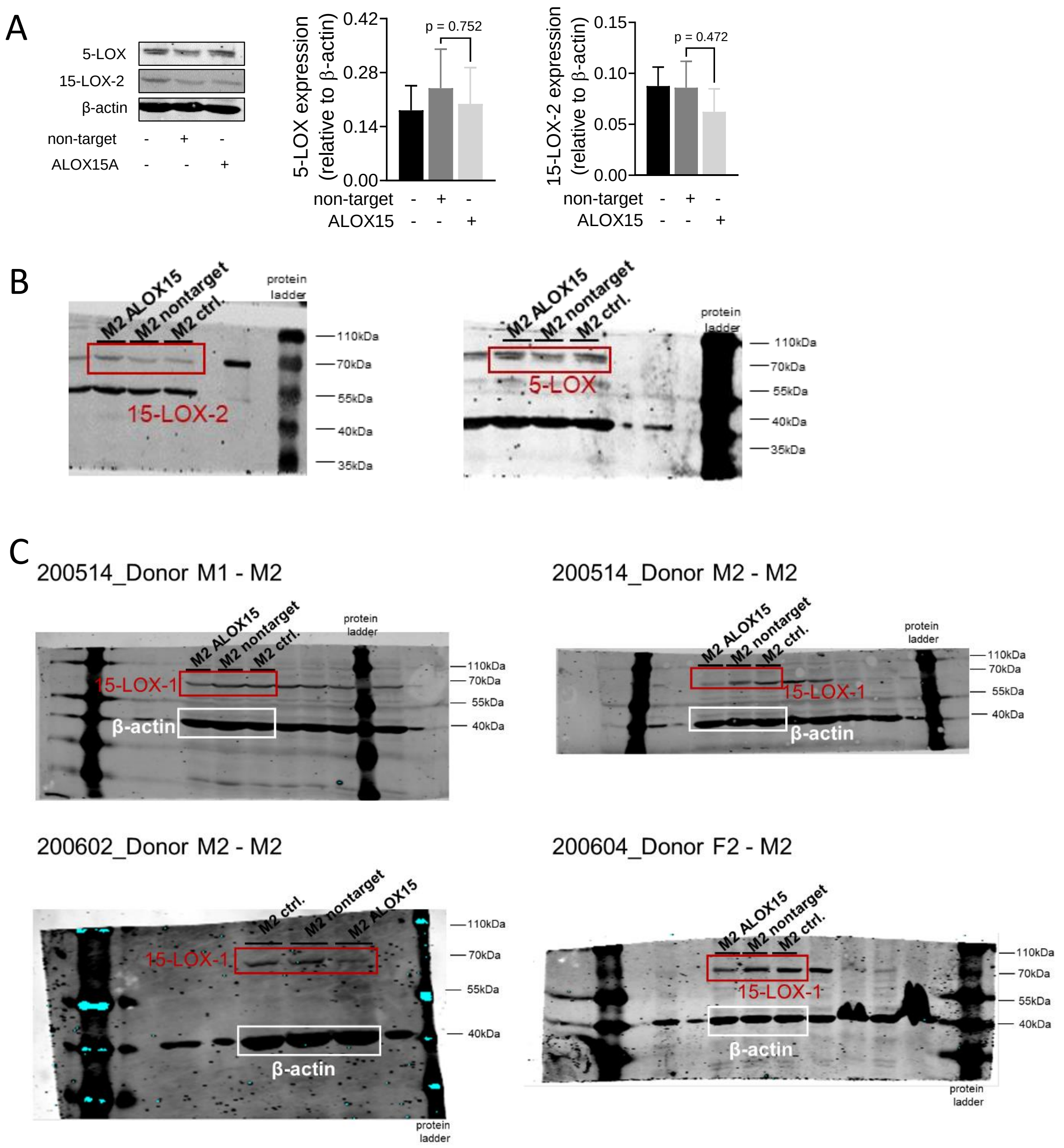

Figure S3. Protein expression of 5-LOX and 15-LOX-2 in ALOX15A siRNA-treated M2-MDMs. (A) Representative Western blot of untreated, non-target or ALOX15 siRNA-transfected M2-MDMs and corresponding densitometric analysis of 5-LOX and 15-LOX-2 protein amounts; n = 4 independent experiments. (B) Single, uncropped Western Blots for 5-LOX and 15-LOX-2 expression; related to Figure S3A. (C) Single, uncropped Western Blots from four independent experiments for 15-LOX-1 expression; related to Figure 3A.

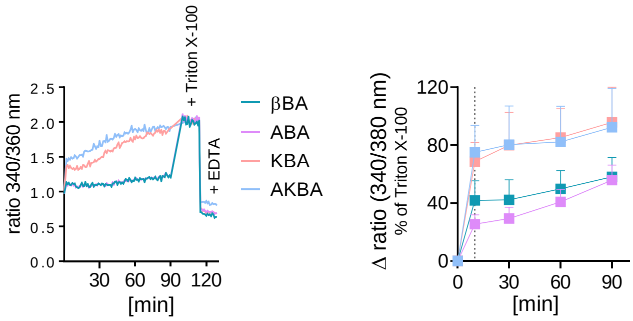

Figure S4. Effects of boswellic acids on $[Ca^{2+}]_i$ in M1-MDMs. Analysis of $[Ca^{2+}]_i$ in Fura-2/AM-loaded M1-MDMs in Krebs-Hepes buffer containing 1 mM Ca^{2+} after stimulation with BAs or vehicle for up to 90 min. Representative line plots are shown as ratio of absorbance at 340/380 nm (left panel) and $[Ca^{2+}]_i$ is given as ration of 340/380 nm in % of maximum $[Ca^{2+}]_i$ determined by cell lysis with triton X-100 (right panel); n = 4 – 5 separate donors.

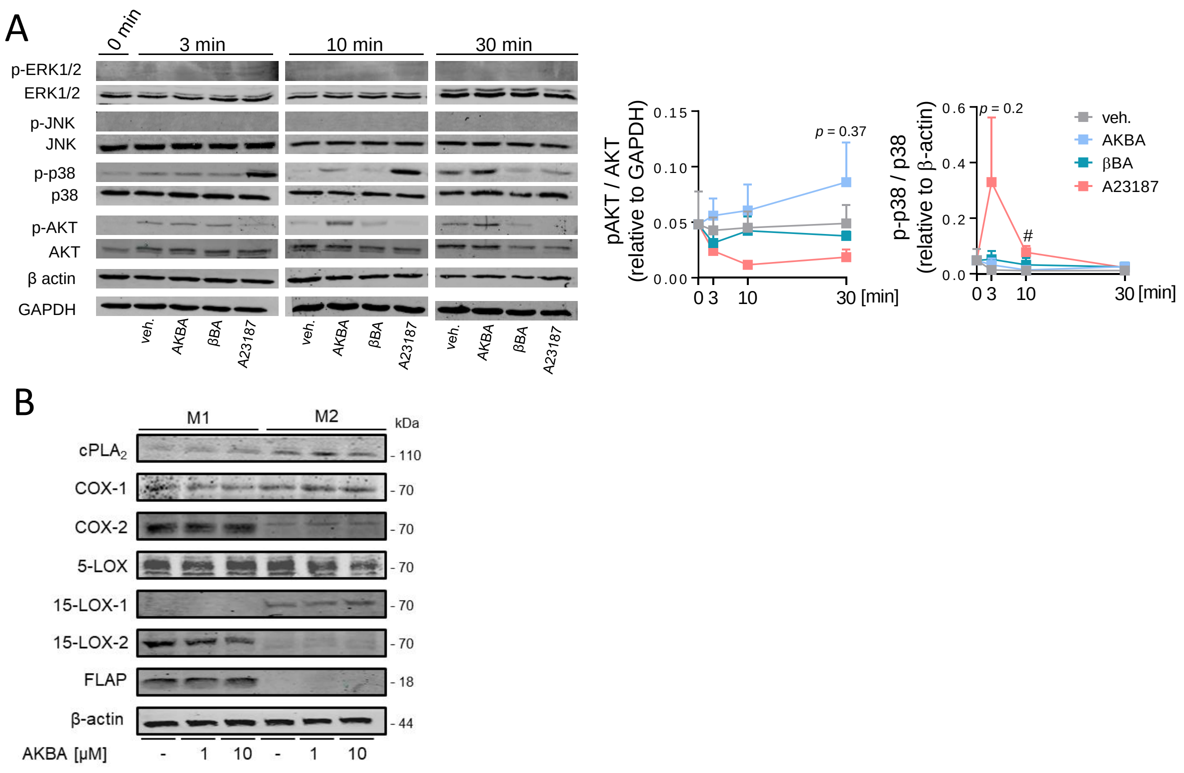

Figure S5. Effect of AKBA on protein kinase activation and on LM-biosynthetic enzyme expression. (A) Western blot analysis of phosphorylated MAPK and Akt in M2-MDMs after treatment with AKBA, βBA (10 µM, each) or A23187 (2.5 µM) for the indicated times. Exemplary plots are shown for each MAPK or Akt, and densitometric analysis of phosphorylation of AKT and p38 MAPK, normalized to β-actin or GAPDH; n = 4-6 separate donors; unpaired Student´s t-test # $p > 0.05$, A23187 versus vehicle. (B) M1- or M2-MDM were incubated with 1 or 10 µM AKBA for 48 h at 37 °C and 5% CO_2. Then cells were harvested and the amounts of the indicated proteins were assessed by Western Blot. Data are representative for 3 independent experiments.

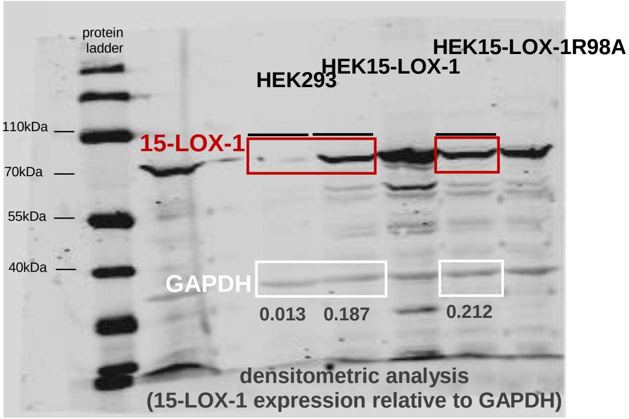

Figure S6. Expression of wt- and R98A-mutant-15-LOX-1 in HEK293 cells at the protein level.

		PMNL		monocytes		M1-like MDMs		M2-like MDMs	
		veh.	AKBA	veh.	AKBA	veh.	AKBA	veh.	AKBA
SPM	PDX	≤ 3	≤ 3	≤ 3	≤ 3	4.3 ± 2.2	7.9 ± 3.9	64 ± 10	177 ± 4.3
	PD1	≤ 3	≤ 3	7.4 ± 2.1	7.6 ± 1.9	5.4 ± 2.1	8.6 ± 3.8	78 ± 10	243 ± 4.1
	MaR1	≤ 3	≤ 3	3.9 ± 1.0	6.1 ± 1.7	≤ 3	≤ 3	34 ± 7.1	48 ± 14
	RvD2	≤ 3	≤ 3	≤ 3	≤ 3	≤ 3	≤ 3	3.0 ± 0.5	6.2 ± 2.0
	RvD5	≤ 3	≤ 3	3.6 ± 1.0	3.8 ± 0.6	≤ 3	4.0 ± 2.2	208 ± 5.2	212 ± 64
	LXA_4	≤ 3	≤ 3	≤ 3	≤ 3	≤ 3	≤ 3	≤ 3	13 ± 2.9
monohydroxylated FA	17-HDHA	3.6 ± 1.6	8.1 ± 2.3	47 ± 9	60 ± 12	259 ± 100	613 ± 269	4395 ± 595	5581 ± 1579
	15-HEPE	≤ 3	≤ 3	4.9 ± 0.7	3.6 ± 0.7	34 ± 14	59 ± 28	783 ± 156	1113 ± 290
	15-HETE	33 ± 16.4	101 ± 1.1	79 ± 4.8	92 ± 10	496 ± 207	1134 ± 506	6592 ± 1003	12493 ± 2976
	14-HDHA	7.5 ± 3.3	17 ± 5.9	1475 ± 385	2373 ± 523	27 ± 6.4	29 ± 3.4	908 ± 140	1606 ± 424
	12-HEPE	3.6 ± 1.2	15 ± 3.6	640 ± 110	932 ± 180	10 ± 3.6	9.1 ± 3.0	132 ± 24	184 ± 47
	12-HETE	95 ± 34.1	304 ± 70	14515 ± 1408	18547 ± 1174	102 ± 44	119 ± 40	714 ± 111	1222 ± 305
	7-HDHA	≤ 3	≤ 3	4.7 ± 0.7	7.9 ± 1.0	16 ± 4.0	19 ± 7.7	179 ± 21	176 ± 43
	4-HDHA	≤ 3	≤ 3	≤ 3	≤ 3	12 ± 2.2	12 ± 2.7	24 ± 7.2	26 ± 6.5
	18-HEPE	≤ 3	≤ 3	4.2 ± 0.5	3.2 ± 0.4	6.9 ± 1.2	4.9 ± 1.1	16 ± 2.6	47 ± 11
COX	PGE_2	3.1 ± 1.1	9.3 ± 4.2	60 ± 5.7	115 ± 7	837 ± 307	897 ± 335	61 ± 14	135 ± 34
	PGD_2	≤ 3	≤ 3	14 ± 2.2	21 ± 1.7	32 ± 8.8	37 ± 10	18 ± 3.5	55 ± 13
	$PGF_{2\alpha}$	≤ 3	≤ 3	26 ± 2.6	14 ± 1.7	193 ± 88	154 ± 62	51 ± 13	34 ± 8.0
	TXB_2	53 ± 17	74 ± 29	7814 ± 1588	2287 ± 525	4070 ± 1575	3613 ± 1552	2769 ± 651	1364 ± 368
5-LOX	5-HEPE	≤ 3	≤ 3	≤ 3	≤ 3	72 ± 27	49 ± 22	36 ± 8.8	29 ± 9.3
	5-HETE	50 ± 2.4	14 ± 3.9	36 ± 3.2	12 ± 1.8	813 ± 269	682 ± 299	298 ± 58	288 ± 71
	t-LTB_4	3.8 ± 1.2	≤ 3	70 ± 17	65 ± 13	110 ± 40	107 ± 61	28 ± 13	34 ± 16
	LTB_4	28 ± 11	6.7 ± 2.2	307 ± 93	87 ± 20	292 ± 150	497 ± 354	29 ± 10	40 ± 12
PUFA	AA	5361 ± 2932	7474 ± 2608	494 ± 37	416 ± 64	12845 ± 957	16345 ± 942	29687 ± 8197	39121 ± 9786
	EPA	207 ± 137	629 ± 228	15 ± 1.8	19 ± 3.9	2345 ± 423	4362 ± 636	8177 ± 3454	13060 ± 4865
	DHA	305 ± 152	279 ± 85	200 ± 40	210 ± 67	9091 ± 3252	9062 ± 3462	22426 ± 6793	25612 ± 8070

Table S1. Modulation of lipid mediator formation in activated 5-LOX-expressing immune cells by AKBA. Human PMNL, monocytes, M1-MDMs and M2-MDMs (10^6 cells, each) were preincubated with 10 µM AKBA or vehicle (0.1% DMSO) for 15 min before stimulation with E. coli (O6:K2:H1; ratio 1:50) for 90 min at 37 °C. Formed LM were isolated from the supernatants by SPE and analyzed by UPLC-MS-MS. Data are means ± S.E.M. given as pg/10^6 cells, n = 3 - 4 , separate donors, each. Relates to Figure 1.

		PMNL		monocytes		M1-like MDMs		M2-like MDMs	
		veh.	AKBA	veh.	AKBA	veh.	AKBA	veh.	AKBA
SPM	PDX	≤ 3	≤ 3	≤ 3	≤ 3	≤ 3	≤ 3	≤ 3	43 ± 18
	PD1	≤ 3	≤ 3	3.8 ± 0.2	6.4 ± 1.6	≤ 3	≤ 3	≤ 3	69 ± 32
	MaR1	≤ 3	≤ 3	≤ 3	3.6 ± 0.1	≤ 3	≤ 3	≤ 3	16 ± 7.8
	RvD2	≤ 3	≤ 3	≤ 3	≤ 3	≤ 3	≤ 3	≤ 3	3.5 ± 1.3
	RvD5	≤ 3	≤ 3	≤ 3	4.0 ± 0.8	≤ 3	≤ 3	≤ 3	85 ± 39
	LXA_4	≤ 3	≤ 3	≤ 3	≤ 3	≤ 3	≤ 3	≤ 3	5.3 ± 1.6
monohydroxylated FA	17-HDHA	≤ 3	6.7 ± 2.1	17 ± 4.7	39 ± 7.6	24 ± 4.0	122 ± 35	61 ± 24	1668 ± 692
	15-HEPE	≤ 3	≤ 3	3.5 ± 0.4	3.5 ± 0.7	9.2 ± 2.5	21 ± 8.0	8.2 ± 2.1	614 ± 340
	15-HETE	4.9 ± 0.5	46 ± 2.0	33 ± 4.3	71 ± 13	42 ± 23	225 ± 57	94 ± 43	6778 ± 3556
	14-HDHA	≤ 3	26 ± 13	237 ± 26	1997 ± 381	18 ± 7.5	21 ± 4.6	16 ± 4.8	558 ± 248
	12-HEPE	≤ 3	11 ± 4.3	264 ± 72	978 ± 171	≤ 3	≤ 3	≤ 3	85 ± 45
	12-HETE	26 ± 6.8	460 ± 220	3952 ± 596	19659 ± 429	33 ± 20	20 ± 4.0	18 ± 5.5	423 ± 210
	7-HDHA	≤ 3	≤ 3	≤ 3	7.3 ± 0.6	4.3 ± 1.6	8.8 ± 2.4	6.9 ± 1.8	67 ± 23
	4-HDHA	≤ 3	≤ 3	≤ 3	≤ 3	3.9 ± 1.7	8.0 ± 4.1	4.7 ± 0.5	20 ± 3.8
	18-HEPE	≤ 3	≤ 3	3.9 ± 0.1	3.1 ± 0.4	3.2 ± 1.3	4.4 ± 1.1	8.7 ± 2.8	35 ± 12
COX	PGE_2	≤ 3	12 ± 4.3	35 ± 3.1	101 ± 7.1	504 ± 159	627 ± 204	6.4 ± 1.6	45 ± 15
	PGD_2	≤ 3	≤ 3	9.5 ± 1.2	19 ± 1.6	20 ± 7.5	29 ± 8.6	3.2 ± 0.5	24 ± 8.4
	$PGF_{2\alpha}$	≤ 3	≤ 3	16 ± 2.0	12 ± 1.6	104 ± 30	120 ± 34	7.7 ± 3.8	9.2 ± 0.4
	TXB_2	31 ± 3.6	64 ± 13	4808 ± 1114	1881 ± 364	1665 ± 560	2145 ± 860	193 ± 65	320 ± 52
5-LOX	5-HEPE	≤ 3	≤ 3	≤ 3	≤ 3	12 ± 7.3	20 ± 7.8	3.5 ± 0.6	13 ± 0.6
	5-HETE	7.2 ± 0.7	8.1 ± 2.1	366 ± 62	196 ± 24.2	215 ± 129	346 ± 182	44 ± 11	116 ± 21
	t-LTB_4	≤ 3	≤ 3	8.5 ± 0.4	39 ± 2.1	30 ± 19	55 ± 29	4.4 ± 0.9	7.6 ± 2.3
	LTB_4	3.3 ± 0.6	≤ 3	10 ± 1.4	37 ± 5.4	70 ± 44	290 ± 190	3.0 ± 0.7	10 ± 5.3
PUFA	AA	1316 ± 379	2274 ± 1053	273 ± 13	752 ± 145	3003 ± 1625	11018 ± 5080	10591 ± 4749	29474 ± 10673
	EPA	94 ± 35	276 ± 167	11 ± 1.1	45 ± 15	220 ± 54	2862 ± 1261	1379 ± 622	10144 ± 3332
	DHA	159 ± 32	140 ± 62	89 ± 11	179 ± 42	2738 ± 1239	7141 ± 5553	8666 ± 3476	22611 ± 738

Table S2. Induction of lipid mediator formation in unstimulated 5-LOX-expressing immune cells by AKBA. Human PMNL, monocytes, M1-MDMs and M2-MDMs (10^6 cells, each) were incubated with 10 µM AKBA or vehicle (0.1% DMSO) for 90 min at 37 °C. Formed LM were isolated by SPE and analyzed by UPLC-MS/MS. Data are means ± S.E.M. given as pg/10^6 cells, n = 4 separate donors, each. Relates to Figure 2.

	untreated	*e. coli*	nontarget + AKBA	ALOX15A + AKBA	-fold
5-HEPE	4.8 ± 0.2	45 ± 17	33 ± 18	27 ± 15	0.8
5-HETE	23 ± 6.6	382 ± 199	266 ± 180	263 ± 185	1.0
t-LTB_4	9.5 ± 1.8	89 ± 24	32 ± 10	17 ± 9.4	0.5
LTB_4	6.7 ± 2.5	76 ± 39	23 ± 14	23 ± 15	1.0
PGE_2	31 ± 20	113 ± 31	107 ± 42	80 ± 40	0.7
PGD_2	4.4 ± 0.2	36 ± 21	48 ± 14	27 ± 6.4	0.6
$PGF_{2\alpha}$	29 ± 5.1	52 ± 8.2	40 ± 3.0	45 ± 7.7	1.1
TXB_2	484 ± 54	1710 ± 146	640 ± 120	857 ± 14	1.3
17-HDHA	101 ± 35	7035 ± 2406	3386 ± 1601	2046 ± 884	0.6
15-HEPE	14 ± 4.9	1230 ± 662	542 ± 239	256 ± 115	0.5
15-HETE	110 ± 57	10745 ± 5456	6648 ± 3101	3477 ± 1643	0.5
14-HDHA	12 ± 3.8	1402 ± 388	945 ± 505	534 ± 325	0.6
12-HEPE	3.7 ± 0.4	180 ± 83	95 ± 40	48 ± 21	0.5
12-HETE	15 ± 3.4	1073 ± 442	532 ± 270	306 ± 178	0.6
7-HDHA	12 ± 1.7	350 ± 91	204 ± 107	128 ± 73	0.6
4-HDHA	7.5 ± 1.0	38 ± 12	38 ± 21	35 ± 21	0.9
18-HEPE	5.0 ± 1.3	26 ± 9.5	42 ± 12	22 ± 5.7	0.5
PDX	≤ 3	23 ± 10	25 ± 15	11 ± 6	0.4
PD1	≤ 3	439 ± 217	109 ± 35	50 ± 19	0.5
MaR1	≤ 3	141 ± 57	23 ± 11	11 ± 6	0.5
RvD2	≤ 3	13 ± 6.1	7.1 ± 1.8	3.4 ± 0.9	0.5
RvD5	≤ 3	705 ± 247	93 ± 34	40 ± 18	0.4
LXA_4	≤ 3	8.2 ± 4.1	15 ± 4.6	8.3 ± 2.0	0.6
AA	5518 ± 1961	42593 ± 13960	51543 ± 29110	61028 ± 33984	1.2
EPA	984 ± 152	7597 ± 3244	13682 ± 7457	17501 ± 9147	1.3
DHA	7534 ± 2965	57336 ± 11875	38259 ± 19758	44323 ± 23488	1.2

Table S3. Effect of AKBA after 15-LOX-1 knockdown in M2 macrophages. Human monocytes-derived macrophages (2 x 10^6 cells) differentiated with M-CSF were transfected with nontarget or ALOX15A siRNA for 48 h during polarization with IL-4 (20 ng/mL). LM production after exposure of macrophages to *E. coli* (ratio 1:50) or AKBA (10 µM) for 180 min, shown as means ± S.E.M. in pg/2 x 10^6 cells and as a heatmap representing –fold change of ALOX15A + AKBA- versus non-target + AKBA-treated cells, n = 4 separate donors.

0 1 70	veh.	AKBA		+ DHA/EPA veh.	+ DHA/EPA AKBA	
5-HEPE	6.2 ± 1.6	14 ± 4.7	2.3	482 ± 184	1044 ± 358	2.2
5-HETE	6.1 ± 1.0	44 ± 17	7.2	28 ± 7	152 ± 35	5.4
t-LTB_4	3.8 ± 1.0	12 ± 4.0	3.1	10 ± 2.9	14 ± 3.7	1.4
LTB_4	5.3 ± 1.8	12 ± 2.8	2.2	7.0 ± 2.7	16 ± 5.8	2.3
PGE_2	28 ± 11	50 ± 11	1.8	24 ± 8	61 ± 14	2.6
PGD_2	12 ± 3.9	24 ± 5.4	2.0	5.4 ± 1.0	16 ± 3.4	3.0
$PGF_{2\alpha}$	84 ± 55	31 ± 5.4	0.4	92 ± 44	61 ± 29	0.7
TXB_2	1680 ± 948	1807 ± 858	1.1	1595 ± 740	1172 ± 393	0.7
17-HDHA	13 ± 0.4	229 ± 18	17.8	79 ± 10	1145 ± 438	14.4
15-HEPE	6.6 ± 2.3	99 ± 8.6	14.9	79 ± 16	2754 ± 1497	34.8
15-HETE	12 ± 1.8	850 ± 231	69.6	25 ± 2.3	1762 ± 563	70.4
14-HDHA	3.6 ± 0.4	83 ± 14	23.0	25 ± 4.3	469 ± 193	18.7
12-HEPE	4.3 ± 1.4	23 ± 4.2	5.4	102 ± 23	980 ± 471	9.6
12-HETE	6.3 ± 1.4	72 ± 25	11.4	15 ± 1.5	115 ± 34	7.4
7-HDHA	14 ± 3.9	38 ± 8.7	2.7	97 ± 20	145 ± 34	1.5
4-HDHA	1.9 ± 0.2	8.8 ± 1.9	4.5	35 ± 11	70 ± 1.9	2.0
18-HEPE	17 ± 4.9	31 ± 12	1.8	2184 ± 610	3280 ± 767	1.5
PD1	≤ 3	5.9 ± 2.5	2.0	≤ 3	12 ± 4.0	3.9
PDX	≤ 3	3.6 ± 0.3	1.2	3.8 ± 0.7	13 ± 2.8	3.4
MaR1	≤ 3	3.8 ± 0.5	1.3	≤ 3	21 ± 9.3	7.1
RvD2	≤ 3	≤ 3	1.0	3.4 ± 0.4	7.1 ± 2.3	2.1
RvD5	≤ 3	22 ± 2.3	7.3	3.4 ± 0.7	94 ± 38	27.5
LXA_4	5.1 ± 1.2	10 ± 3.7	2.0	9 ± 2.1	14 ± 3.8	1.6
AA	9058 ± 4157	102130 ± 47345	11.3	15055 ± 5385	171493 ± 73840	11.4
EPA	6421 ± 2154	27951 ± 10876	4.4	147195 ± 42815	357772 ± 37365	2.4
DHA	2165 ± 869	11365 ± 4595	5.2	17053 ± 4450	47005 ± 5827	2.8

Table S4. Effect of AKBA on M2-MDMs after DHA/EPA supplementation. M2-MDMs where co-incubated with 3 µg/mL of a DHA- and EPA-enriched fatty acid supplementary plus AKBA (10 µM) or vehicle (0.1% DMSO) for 180 min at 37°C. Formed LM were isolated by SPE and analyzed by UPLC-MS/MS. Data are shown as mean ± S.E.M. in pg/2 x 10^6 cells and as a heatmap representing the −fold change of AKBA- versus vehicle-treated cells, n = 4 separate donors.

0 1 180	+ Ca^{2+} veh.	+ Ca^{2+} + AKBA		+ EDTA veh.	+ EDTA + AKBA		+ EDTA/BAPTA-AM veh.	+ EDTA/BAPTA-AM + AKBA	
5-HEPE	7.4 ± 1.7	30 ± 12	4.1	7.8 ± 2.1	22 ± 6.8	2.7	8.9 ± 2.2	18 ± 4.4	2.0
5-HETE	13 ± 3.8	160 ± 78	12.5	33 ± 16	131 ± 55	4.0	35 ± 13	109 ± 43	3.1
t-LTB_4	15 ± 3.3	30 ± 8.7	2.0	17 ± 2.2	34 ± 15	2.0	12 ± 1.0	35 ± 21	3.0
LTB_4	12 ± 2.4	22 ± 7.5	1.9	18 ± 5.7	14 ± 2.1	0.8	12 ± 2.2	14 ± 4.2	1.1
PGE_2	54 ± 33	100 ± 3.0	1.9	30 ± 11	94 ± 31	3.2	28 ± 9.2	138 ± 51	5.0
PGD_2	4.0 ± 1.7	26 ± 11	6.6	3.1 ± 0.5	31 ± 17	9.9	6.3 ± 3.0	37 ± 22	5.8
$PGF_{2\alpha}$	16 ± 1.2	17 ± 3.6	1.0	17 ± 2.5	23 ± 2.9	1.4	16 ± 0.7	32 ± 4.4	2.0
TXB_2	663 ± 241	722 ± 315	1.1	459 ± 100	498 ± 72	1.1	453 ± 159	1064 ± 413	2.3
17-HDHA	43 ± 10	1649 ± 561	38.5	49 ± 6.1	1387 ± 487	28.4	183 ± 62	1680 ± 862	9.2
15-HEPE	7.8 ± 1.1	336 ± 108	43.1	8.5 ± 0.4	296 ± 117	34.7	17 ± 4.6	297 ± 179	17.8
15-HETE	25 ± 3.5	4544 ± 1779	179.3	47 ± 10	3778 ± 1751	80.8	107 ± 32	4171 ± 2775	38.9
14-HDHA	7.6 ± 2.6	444 ± 145	58.0	10 ± 1.3	460 ± 198	44.0	15 ± 5.0	536 ± 323	35.8
12-HEPE	3.0 ± 0.8	53 ± 16	17.9	2.7 ± 0.2	51 ± 18	19.3	4.4 ± 1.6	51 ± 29	11.6
12-HETE	12 ± 2.8	256 ± 95	21.2	14 ± 3.9	262 ± 126	18.6	17 ± 3.7	266 ± 153	15.5
7-HDHA	9.0 ± 0.3	71 ± 20	7.9	11 ± 2.0	76 ± 27	6.7	11 ± 1.9	93 ± 46	8.6
4-HDHA	7.7 ± 2.3	26 ± 9.1	3.4	7.6 ± 2.2	26 ± 9.1	3.4	13 ± 4.2	19 ± 2.3	1.5
PDX	≤ 3	2.8 ± 1.3	0.9	≤ 3	≤ 3	1.0	≤ 3	≤ 3	1.0
PD1	≤ 3	17 ± 6.3	5.5	≤ 3	16 ± 7.4	5.4	≤ 3	20 ± 14	6.8
MaR1	≤ 3	12 ± 7.4	4.1	≤ 3	15 ± 11	5.2	≤ 3	24 ± 19	8.1
RvD2	≤ 3	≤ 3	1.0	≤ 3	≤ 3	1.0	≤ 3	≤ 3	1.0
RvD5	≤ 3	79 ± 43	26.5	≤ 3	91 ± 55	30.4	≤ 3	29 ± 19	9.6
LXA_4	7.3 ± 1.8	7.9 ± 0.9	1.1	7.7 ± 3.6	3.1 ± 0.5	0.4	6.3 ± 1.5	8.7 ± 3.2	1.4
AA	151888 ± 55602	686514 ± 380906	4.5	132677 ± 58023	683429 ± 400372	5.2	277182 ± 140667	943634 ± 333106	3.4
EPA	40730 ± 13045	235471 ± 128111	5.8	32783 ± 14006	227232 ± 136306	6.9	64814 ± 30199	304053 ± 135018	4.7
DHA	29251 ± 12170	70464 ± 32619	2.4	10198 ± 2537	76432 ± 38459	7.5	60299 ± 27902	114367 ± 34624	1.9

Table S5. 15-LOX-1 activation by AKBA in M2-MDMs is insensitive to Ca^{2+}. Human M2-MDM were preincubated 20 min with PBS containing Ca^{2+} (1 mM), EDTA (0.5 mM) or EDTA plus BAPTA-AM (20 µM) before cells were stimulated with AKBA (10 µM) or vehicle (0.1% DMSO) for 180 min at 37°C. Formed LM were isolated by SPE and analyzed by UPLC-MS-MS. Data are shown as mean ± S.E.M. in pg/2 x 10^6 MDM and as a heatmap representing the –fold change of AKBA- versus vehicle-treated cells; n = 3 independent experiments.

0 1 5	veh.	AKBA									
			skepinone L		U0126		SP600125		LY 294002		staurosporine	
5-HEPE	8.3 ± 3.8	26 ± 2.9	23 ± 4.5	0.9	17 ± 1.0	0.7	45 ± 1.9	1.8	28 ± 2.6	1.1	30 ± 2.9	1.2
5-HETE	13 ± 5.8	149 ± 17	124 ± 21	0.8	58 ± 16	0.4	273 ± 29	1.8	158 ± 16	1.1	157 ± 29	1.1
t-LTB_4	6.6 ± 1.2	12 ± 1.3	12 ± 2.8	1.0	7.9 ± 2.2	0.7	23 ± 3.2	2.0	13 ± 1.4	1.1	12 ± 0.4	1.0
LTB_4	3.6 ± 0.5	8.5 ± 1.0	11 ± 1.8	1.3	5.6 ± 1.0	0.7	14 ± 1.5	1.6	8.4 ± 2.1	1.0	8.3 ± 0.6	1.0
PGE_2	12 ± 5.3	42 ± 6.8	46 ± 10	1.1	46 ± 16	1.1	43 ± 8.7	1.0	42 ± 6.7	1.0	46 ± 10	1.1
PGD_2	6.3 ± 1.8	19 ± 6.5	20 ± 10	1.0	18 ± 10	0.9	17 ± 4.6	0.9	16 ± 4.4	0.8	19 ± 8.3	1.0
$PGF_{2\alpha}$	30 ± 8.7	45 ± 16	45 ± 16	1.0	48 ± 15	1.1	40 ± 10	0.9	38 ± 13	0.8	40 ± 12	0.9
TXB_2	895 ± 503	1407 ± 649	1291 ± 634	0.9	1212 ± 684	0.9	1082 ± 443	0.8	1179 ± 456	0.8	1274 ± 599	0.9
17-HDHA	14 ± 4.5	455 ± 134	523 ± 278	1.1	523 ± 342	1.1	738 ± 339	1.6	454 ± 148	1.0	784 ± 425	1.7
15-HEPE	6.9 ± 2.2	46 ± 18	61 ± 42	1.3	75 ± 54	1.6	92 ± 53	2.0	51 ± 24	1.1	84 ± 54	1.8
15-HETE	14 ± 4.8	687 ± 217	726 ± 431	1.1	962 ± 694	1.4	1170 ± 528	1.7	692 ± 266	1.0	1066 ± 585	1.6
14-HDHA	2.6 ± 1.0	100 ± 33	135 ± 86	1.4	139 ± 96	1.4	177 ± 80	1.8	88 ± 40	0.9	155 ± 88	1.5
12-HEPE	4.9 ± 2.8	17 ± 3.5	22 ± 11	1.3	23 ± 9.4	1.3	29 ± 7.9	1.7	15 ± 2.9	0.9	21 ± 7.9	1.2
12-HETE	4.8 ± 1.2	78 ± 2.6	96 ± 34	1.2	92 ± 31	1.2	132 ± 25	1.7	75 ± 7.7	1.0	89 ± 26	1.1
7-HDHA	1.7 ± 0.1	33 ± 6.8	28 ± 3.0	0.9	18 ± 2.7	0.6	58 ± 7.0	1.8	31 ± 4.1	1.0	34 ± 1.2	1.1
4-HDHA	4.3 ± 1.2	39 ± 6.3	38 ± 10	1.0	26 ± 6.2	0.7	60 ± 3.8	1.6	38 ± 4.9	1.0	38 ± 6.8	1.0
18-HEPE	15 ± 3.7	23 ± 5.2	19 ± 7.9	0.8	21 ± 2.8	0.9	45 ± 1.6	1.9	24 ± 3.8	1.0	31 ± 7.5	1.0
PDX	≤3	≤3	≤3	1.0	≤3	1.0	4.6 ± 2.6	1.5	≤3	1.0	≤3	1.0
PD1	≤3	5.0 ± 1.6	6.5 ± 3.6	1.3	6.3 ± 4.3	1.3	8.4 ± 2.9	1.7	5.0 ± 1.9	1.0	6.8 ± 3.2	1.3
MaR1	≤3	≤3	4.0 ± 2.8	1.3	≤3	1.0	5.7 ± 4.0	1.9	≤3	1.0	3.4 ± 2.2	1.1
RvD2	≤3	≤3	≤3	1.0	≤3	1.0	≤3	1.0	≤3	1.0	≤3	1.0
RvD5	≤3	13 ± 7.1	21 ± 16	1.6	23 ± 19	1.8	35 ± 24	2.6	12 ± 7.5	0.9	25 ± 19	1.9
LXA_4	≤3	≤3	≤3	1.2	≤3	1.0	≤3	1.0	≤3	1.0	≤3	1.0
AA	58918 ± 9762	1310641 ± 126069	1610951 ± 160055	1.2	1638383 ± 138815	1.3	1644521 ± 166411	1.3	1417700 ± 112040	1.1	1549322 ± 89730	1.2
EPA	44611 ± 6616	428833 ± 42052	572284 ± 90068	1.3	563944 ± 16119	1.3	627652 ± 63377	1.5	505973 ± 66680	1.2	588311 ± 51272	1.4
DHA	18847 ± 6594	228971 ± 13289	263136 ± 3700	1.1	276276 ± 14946	1.2	277151 ± 16747	1.2	250135 ± 18990	1.1	271821 ± 5850	1.2

Table S6. Effects of kinase inhibitors on AKBA-induced LM formation in M2-MDM. M2-MDMs (2 x 10^6) were pre-treated with the respective kinase inhibitors as follows: 1 µM skepinone-L, 3 µM U0126, 10 µM SP600125; 3 µM LY-294002, and 1 µM staurosporine. After 10 min at 37 °C, 10 µM AKBA were added and the cells were further incubated for 180 min at 37°C. Formed LM were isolated from the supernatants by SPE and analyzed by UPLC-MS-MS. Data are given as mean ± S.E.M. in pg/ 10^6 cells and shown in a heatmap representing the –fold change of stimulus-treated vs. vehicle-treated M2-MDM, n = 3 – 7, separate donors.

0 1 70	293			5-LOX/FLAP			12-LOX			15-LOX-1			15-LOX-2		
	veh.	AKBA	Fold	veh.	AKBA	Fold	veh.	AKBA	Fold	veh.	AKBA	Fold	veh.	AKBA	Fold
5-HEPE	53 ± 50	41 ± 30	0.8	786 ± 135	3690 ± 1003	4.7	46 ± 40	63 ± 57	1.4	3.4 ± 1.2	6.0 ± 0.7	1.8	39 ± 38	57 ± 46	1.5
5-HETE	175 ± 132	74 ± 25	0.4	345 ± 29	19198 ± 5913	55.7	88 ± 30	240 ± 213	2.7	5.0 ± 1.5	12 ± 1.1	2.5	40 ± 20	51 ± 8.1	1.3
t-LTB_4	12 ± 7.0	8.5 ± 3.2	0.7	361 ± 74	1733 ± 449	4.8	24 ± 11	84 ± 30	3.5	2.3 ± 0.8	2.2 ± 0.1	0.9	18 ± 11	12 ± 1.3	0.6
LTB_4	12 ± 7.9	4.5 ± 1.3	0.4	437 ± 88	3953 ± 882	9.0	10 ± 1.8	30 ± 10	3.2	3.9 ± 0.3	4.8 ± 0.7	1.2	4.9 ± 2.5	7.7 ± 1.2	1.6
5,15-diHETE	10 ± 2.7	3.9 ± 0.9	0.4	233 ± 51	513 ± 199	2.2	8.6 ± 2.3	22 ± 8.6	2.5	16 ± 9	19 ± 3.8	1.2	16 ± 1.2	72 ± 18	4.6
PGE_2	2.5 ± 0.1	6.6 ± 3.1	2.6	44 ± 14	56 ± 30	1.3	8.1 ± 3.0	10 ± 2.8	1.2	0.9 ± 0.3	0.4 ± 0.1	0.4	9.1 ± 1.1	11 ± 0.6	1.2
PGD_2	0.5 ± 0.1	1.1 ± 0.4	2.0	37 ± 3.4	78 ± 27	2.1	1.3 ± 0.3	1.2 ± 0.6	0.9	6.5 ± 4.9	4.1 ± 0.1	0.6	0.8 ± 0.2	1.4 ± 0.4	1.8
17-HDHA	23 ± 11	20 ± 3.6	0.9	570 ± 128	1625 ± 637	2.9	42 ± 21	56 ± 23	1.3	10 ± 3.7	675 ± 33	64.9	2898 ± 905	20781 ± 1795	7.2
15-HEPE	6.7 ± 2.6	5.6 ± 1.1	0.8	134 ± 32	183 ± 43	1.4	11 ± 4.4	16 ± 3.1	1.5	3.7 ± 1.1	198 ± 27	52.9	1233 ± 95	6594 ± 761	5.3
15-HETE	40 ± 22	54 ± 27	1.4	578 ± 148	965 ± 290	1.7	24 ± 1.8	63 ± 27	2.6	9.4 ± 1.3	682 ± 88	72.9	2326 ± 861	26734 ± 3923	11.5
14-HDHA	14 ± 9.1	21 ± 3.2	1.5	146 ± 74	563 ± 115	3.8	4195 ± 549	16730 ± 5642	4.0	7 ± 1.0	497 ± 15	68.4	31 ± 10	178 ± 27	5.7
12-HEPE	8.3 ± 3.7	9.0 ± 2.2	1.1	149 ± 63	505 ± 140	3.4	2563 ± 507	13281 ± 3630	5.2	2.5 ± 0.2	55 ± 6	21.9	15 ± 2.0	32 ± 2.4	2.1
12-HETE	35 ± 16	42 ± 10	1.2	352 ± 173	4807 ± 2247	13.7	7253 ± 1138	39504 ± 11166	5.4	9 ± 3.7	134 ± 9	14.5	2046 ± 2019	1504 ± 1427	0.7
7-HDHA	20 ± 6	15 ± 5.7	0.8	962 ± 344	4723 ± 1478	4.9	11 ± 1.8	19 ± 1.6	1.6	5.7 ± 1.2	41 ± 1.7	7.3	10 ± 1.9	10 ± 0.9	1.0
4-HDHA	≤ 3	7.5 ± 0.1	2.5	213 ± 93	276 ± 64	1.3	≤ 3	≤ 3	1.0	4.2 ± 1.2	8.1 ± 1.6	1.9	≤ 3	5.8 ± 0.9	1.9
18-HEPE	16 ± 14	15 ± 8.5	0.9	398 ± 145	353 ± 127	0.9	28 ± 7.8	25 ± 2.5	0.9	2.8 ± 0.3	12 ± 2.4	4.1	7.7 ± 3.6	31 ± 3.6	4.0
AA	120336 ± 39852	228433 ± 121856	1.9	88925 ± 16252	847855 ± 457842	9.5	34924 ± 14133	128062 ± 61946	3.7	5920 ± 1107	68837 ± 19180	11.6	46554 ± 15760	306068 ± 181650	6.6
EPA	72283 ± 38292	152781 ± 68461	2.1	21226 ± 3279	138723 ± 58492	6.5	30005 ± 23728	40336 ± 14753	1.3	2410 ± 1392	57642 ± 8676	23.9	43817 ± 29806	140321 ± 66332	3.2
DHA	21768 ± 9816	45620 ± 15458	2.1	88455 ± 2130	287836 ± 88630	3.3	10988 ± 4895	14919 ± 2133	1.4	4519 ± 1135	42512 ± 1575	9.4	15821 ± 3970	32981 ± 10402	2.1

Table S7. Effects of AKBA on LM formation in LOX-transfected HEK 293 cells. 10^6 HEK293 cells stably transfected with human recombinant LOXs (as indicated) were incubated with vehicle (0.1% DMSO) or AKBA (25 µM) for 180 min in PG buffer plus 1 mM Ca^{2+}. Cell supernatant was subjected to SPE and UPLC-MS/MS analysis. LM formation is shown as pg/10^6 cells, given as mean ± S.E.M., and as a heatmap representing the –fold increase, for n = 3 independent experiments.

	15-LOX1 wt						15-LOX-1_R98A								
	veh			AKBA			veh				AKBA				
17-HDHA	16	±	9	372	±	161	17	±	10	1.1	58	±	12	0.2
15-HEPE	266	±	102	6748	±	1167	171	±	49	0.6	1162	±	161	0.2
15-HETE	259	±	55	2255	±	280	252	±	68	1.0	1026	±	184	0.5
14-HDHA	13	±	8.3	214	±	81	11	±	5.7	0.8	45	±	7.2	0.2
12-HEPE	126	±	27	2056	±	324	119	±	33	0.9	544	±	86	0.3
12-HETE	58	±	6.0	478	±	44	61	±	17	1.1	250	±	40	0.5
7-HDHA	12	±	4.1	42	±	18	11	±	5.4	1.0	17	±	3.8	0.4
4-HDHA	14	±	7.4	23	±	1.8	14	±	8.4	1.0	27	±	1.3	1.2
18-HEPE	590	±	126	1882	±	213	666	±	209	1.1	2050	±	373	1.1
5-HEPE	314	±	64	1470	±	230	372	±	139	1.2	1661	±	375	1.1
5-HETE	215	±	96	485	±	84	129	±	37	0.6	538	±	120	1.1
t-LTB_4	3.5	±	0.6	5.9	±	1.3	4.3	±	0.8	1.2	4.9	±	0.9	0.8
LTB_4	5.3	±	0.2	10	±	1.3	6	±	0.8	1.2	9.0	±	1.5	0.9
PGD_2	10	±	0.7	11	±	2.0	12	±	3.0	1.2	10	±	1.5	0.9
PGE_2	33	±	15	31	±	2.8	37	±	15	1.1	24	±	3.1	0.8
PD1	19	±	7	36	±	16	16	±	4.6	0.9	32	±	15	0.9
PDX	34	±	22	8.7	±	1.5	33	±	21	1.0	7.4	±	2.8	0.8
RvD5	3.8	±	1.3	13	±	6.8		≤3		0.8		≤3		0.2
MaR1	14	±	3.1	25	±	3.7	28	±	16	2.0	16	±	3.6	0.6
5,15-diHETE	27	±	7.8	22	±	4.4	34	±	11	1.3	18	±	6.5	0.8

Heatmap scale: 0 1 2

Table S8. Effects of AKBA on mutated 15-LOX-1 (R98A) in stable transfected HEK 293 cells. 10^6 stable transfected HEK293 cells (wt- or R98A mutant, as indicated) were incubated with vehicle (0.1% DMSO) or AKBA (10 µM) for 180 min in PG buffer plus 1 mM Ca^{2+}. Cell supernatant was subjected to SPE and UPLC-MS/MS analysis. LM formation is given as mean ± S.E.M. in pg/ 10^6 cells; n = 3 independent experiments. Data are shown as pg/10^6 cells and as a heatmap representing the –fold change of vehicle- or AKBA-treated cells versus cells expressing R98A mutant-15-LOX-1 or wt-15-LOX-1, respectively.
